# Functional characterization of two flavone synthase II members in citrus

**DOI:** 10.1093/hr/uhad113

**Published:** 2023-05-31

**Authors:** Juan Zheng, Chenning Zhao, Zhenkun Liao, Xiaojuan Liu, Qin Gong, Chenwen Zhou, Yilong Liu, Yue Wang, Jinping Cao, Lili Liu, Dengliang Wang, Chongde Sun

**Affiliations:** Plant Growth, Development and Quality Improvement, Zhejiang Provincial Key Laboratory of Integrative Biology of Horticultural Plants, Zhejiang University, Hangzhou, 310000, China; Plant Growth, Development and Quality Improvement, Zhejiang Provincial Key Laboratory of Integrative Biology of Horticultural Plants, Zhejiang University, Hangzhou, 310000, China; Plant Growth, Development and Quality Improvement, Zhejiang Provincial Key Laboratory of Integrative Biology of Horticultural Plants, Zhejiang University, Hangzhou, 310000, China; Plant Growth, Development and Quality Improvement, Zhejiang Provincial Key Laboratory of Integrative Biology of Horticultural Plants, Zhejiang University, Hangzhou, 310000, China; Plant Growth, Development and Quality Improvement, Zhejiang Provincial Key Laboratory of Integrative Biology of Horticultural Plants, Zhejiang University, Hangzhou, 310000, China; Plant Growth, Development and Quality Improvement, Zhejiang Provincial Key Laboratory of Integrative Biology of Horticultural Plants, Zhejiang University, Hangzhou, 310000, China; Plant Growth, Development and Quality Improvement, Zhejiang Provincial Key Laboratory of Integrative Biology of Horticultural Plants, Zhejiang University, Hangzhou, 310000, China; Plant Growth, Development and Quality Improvement, Zhejiang Provincial Key Laboratory of Integrative Biology of Horticultural Plants, Zhejiang University, Hangzhou, 310000, China; Plant Growth, Development and Quality Improvement, Zhejiang Provincial Key Laboratory of Integrative Biology of Horticultural Plants, Zhejiang University, Hangzhou, 310000, China; Quzhou Academy of Agriculture and Forestry Science, Quzhou, 324000, China; Quzhou Academy of Agriculture and Forestry Science, Quzhou, 324000, China; Plant Growth, Development and Quality Improvement, Zhejiang Provincial Key Laboratory of Integrative Biology of Horticultural Plants, Zhejiang University, Hangzhou, 310000, China

## Abstract

Polymethoxylated flavones (PMFs), the main form of flavones in citrus, are derived from the flavone branch of the flavonoid biosynthesis pathway. Flavone synthases (FNSs) are enzymes that catalyze the synthesis of flavones from flavanones. However, the FNS in citrus has not been characterized yet. Here, we identified two type II FNSs, designated CitFNSII-1 and CitFNSII-2, based on phylogenetics and transcriptome analysis. Both recombinant CitFNSII-1 and CitFNSII-2 proteins directly converted naringenin, pinocembrin, and liquiritigenin to the corresponding flavones in yeast. In addition, transient overexpression of *CitFNSII-1* and *CitFNSII-2*, respectively, in citrus peel significantly enhanced the accumulation of total PMFs, while virus-induced *CitFNSII-1* and *CitFNSII-2* genes silencing simultaneously significantly reduced the expression levels of both genes and total PMF content in citrus seedlings. *CitFNSII-1* and *CitFNSII-2* presented distinct expression patterns in different cultivars as well as different developmental stages. Methyl salicylate (MeSA) treatment reduced the *CitFNSII-2* expression as well as the PMFs content in the peel of *Citrus sinensis* fruit but did not affect the *CitFNSII-1* expression. These results indicated that both CitFNSII-1 and CitFNSII-2 participated in the flavone biosynthesis in citrus while the regulatory mechanism governing their expression might be specific. Our findings improved the understanding of the PMFs biosynthesis pathway in citrus and laid the foundation for further investigation on flavone synthesis regulation.

## Introduction

Citrus, as one of the most cultivated fruits in the world, is favored by consumers for its delicious flavor and abundance of nutritional components, such as carotenoids, vitamin C, folate, dietary fiber, and flavonoids [1]. Flavonoids are one type of the most important bioactive substances in citrus. They are largely distributed in the peel and pulp of citrus fruits and confer significant pharmacological benefits for human health. As the ubiquitous secondary metabolites in the plant kingdom, flavonoids can be classified into several subgroups on the basis of their molecular structures [2]. Flavanones, flavones, flavonols, and anthocyanidins have been identified from *Citrus* genus, among which flavanones and flavones are the predominant components [3]. Polymethoxylated flavones (PMFs), named for their highly *O*-methylated structures, are the main forms of flavones presenting in citrus [4], with sinensetin, nobiletin, tangeretin, and 5-demethylnobiletin as the representative compounds [5]. Previous researches indicated that PMF accumulation varied among citrus varieties. Mandarins (*Citrus reticulata*) and sweet oranges (*Citrus sinensis*) accumulated a high amount of PMFs in their flavedo [6, 7]. A large number of *in vivo* and *in vitro* studies proved that the special structure of PMFs endows them with higher biological activity than other flavonoids and PMFs in citrus have a good potential for drug development [8]. Therefore, it is of great significance to elucidate the biosynthetic pathway of PMFs in citrus for increasing the production and application of these bioactive compounds.

**Figure 1:**
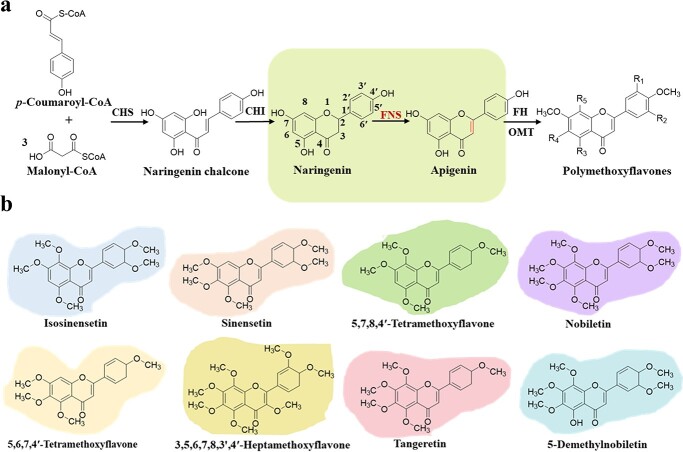
PMFs in citrus plants and their biosynthesis pathway. **a** The PMFs biosynthesis pathway in citrus plants. CHS, chalcone synthase; CHI, chalcone isomerase; FH, flavonoid hydroxylase; FNS, flavone synthase; OMT, *O*-methyltransferase. **b** Structures of representative citrus PMFs.

PMFs in citrus are derived from the flavone branch of the flavonoid biosynthetic pathway. Flavanones, synthesized from stepwise reactions of chalcone synthase (CHS) and chalcone isomerase (CHI), are catalyzed by flavone synthase (FNS) to generate corresponding flavones, followed by hydroxylation and methylation modifications at multiple sites of flavone skeleton, resulting in diverse structures of PMFs (Fig. 1a and b). In this process, FNS is the key enzyme responsible for the substrates entering the flavone synthesis branch [9], and flavonoid hydroxylase (FH) and flavonoid *O*-methyltransferase (OMT) play the role for modifications. However, the genes encoding the pivotal FNS in citrus have not been characterized yet.

FNS belongs to oxidases and it converses flavanones to flavones by introducing a C-C double bond between the 2–3 positions of the flavan skeleton. There are two distinct types of FNS (FNSI and FNSII) that have been reported to participate in the process [9]. In addition, a polyphenol oxidase in tomato exhibiting FNS-like activity has been identified recently [10]. FNSI belongs to the Fe^2+^/2-oxoglutarate-dependent dioxygenase (2-OGDD) family, and is a soluble protein located in the cytoplasm. It is mainly studied in *Apiaceae* species [11], and has been identified in Arabidopsis (*Arabidopsis thaliana*), rice (*Oryza sativa*), moss (*Pohlia nutans*), and mulberry (*Morus alba*) lately [12–15]. Unlike FNSI, FNSII is a membrane-bound protein and belongs to the NADPH-dependent cytochrome P450 (CYP450) monooxygenase family. In addition, the distribution of FNSII is more ubiquitous in plants than FNSI. At present, almost all the FNSII proteins identified in plants belong to the CYP93 subfamily and there is also a class of F2H in this family, which can catalyze the hydroxylation of flavanones to form 2-OH flavanones, providing the basis for the further introduction of *C*-glycosides [16, 17]. The characterized FNSIIs in dicots mainly belong to the CYP93B subfamily, such as *Medicago truncatula* (CYP93B10 and CYP93B11) and *Glycine max* (CYP93B16) [18, 19]; while the identified FNSIIs in monocots belong to the CYP93G subfamily, such as *Sorghum bicolor* (CYP93G3) and *Zea mays* (CYP93G7) [20, 21]. Most FNSIIs can catalyze the formation of double bonds in flavones, but some FNSIIs also have F2H activity, such as GeFNSII (*Glycyrrhiza echinata*) and MtFNSIIs (*M. truncatula*) [19, 22]. Additionally, FNSII is proven to be an important composition of flavonoid metabolon because of its membrane localization [23]. In recent years, an increasing number of FNSIIs have been successfully identified and analysed, but they are mainly concentrated in model plants, cereal crops, or medicinal plants such as *Antirrhinum maju*，*O. sativa*, and *Scutellaria baicalensis* [17, 24, 25]. Among them, FNSII in *S. baicalensis* is also reported to be related to the root-specific flavone synthesis, indicating that different FNSII members might be involved in the formation of different types of flavones [24]. There are also some FNSIIs reported in ornamental plants, such as *Chrysanthemum morifolium*, *Torenia spp.* and *Dahlia variabilis* [26–28], as flavone is also an important co-pigment. Recently, two FcFNSIIs have been identified from kumquat (*Fortunella crassifolia*), a related species of citrus. However, because the accumulation patterns and components of flavones are completely different between kumquat and citrus, there might be other FNSII members in citrus involved in PMFs synthesis.

Many biotic and abiotic factors affect the synthesis of flavonoids, among which phytohormones play an important role [29, 30]. Postharvest treatments with γ-aminobutyric acid (GABA), methyl jasmonate (MeJA) or methyl salicylate (MeSA) are proved to be effective in preserving the bioactive compounds and nutritional quality of blood oranges [31]. Studies have proved that MeJA treatment increased the content of flavones as well as the expression levels of *SbFNSII-2* in the root cultures of *S. baicalensis* [24]. Previous studies indicated that citrus flavonoids can effectively protecting citrus fruits from pathogenic attack, such as *Colletotrichum gloeosporioides* and *Penicillum digitatum* [32, 33]. Salicylic acid (SA) and its methyl ester, MeSA, are common inducers of plant defense responses. They were widely used to study resistance caused by postharvest pathogens in citrus [34, 35]. In addition, it was also shown that exogenous SA treatment induced enhanced resistance to *Penicillium fingerum* in citrus fruits may be related to the action of MeSA [35]. Studies have shown that the treatment of SA could increase the content of eriocitrin, narirutin, poncirin, and *β*-cryptoxanthin in the juice sacs of satsuma mandarin [36]. MeSA was also proved to enhance flavonoid biosynthesis in tea leaves by stimulating the phenylpropanoid pathway [37]. The influence of MeSA on the biosynthesis of citrus flavone is still unclear, and whether it has a regulatory effect on FNS expression needs further study.

In this study, we identified two FNSIIs belonging to CYP93B from citrus, and termed as CitFNSII-1 and CitFNSII-2, respectively. Sequence alignment showed that *CitFNSII-2* shared 95.63% identity with *CitFNSII-1* at nucleotide level, suggesting that they might derive from gene duplication. The results of *in vivo* yeast enzyme assays, transient overexpression and virus-induced gene silencing in citrus illustrated that CitFNSII-1 and CitFNSII-2 play important but similar roles in citrus flavone synthesis, while specific expression patterns of these two genes and results of MeSA treatment indicated that they might be regulated by different mechanisms. This is the first functional characterization of citrus FNS enzymes, and the findings not only improved the understanding on PMFs biosynthesis pathway in citrus but also revealed function and expression differences of two *FNS* gene copies.

## Results

### Identification of the candidate genes encoding FNSII in citrus

**Figure 2:**
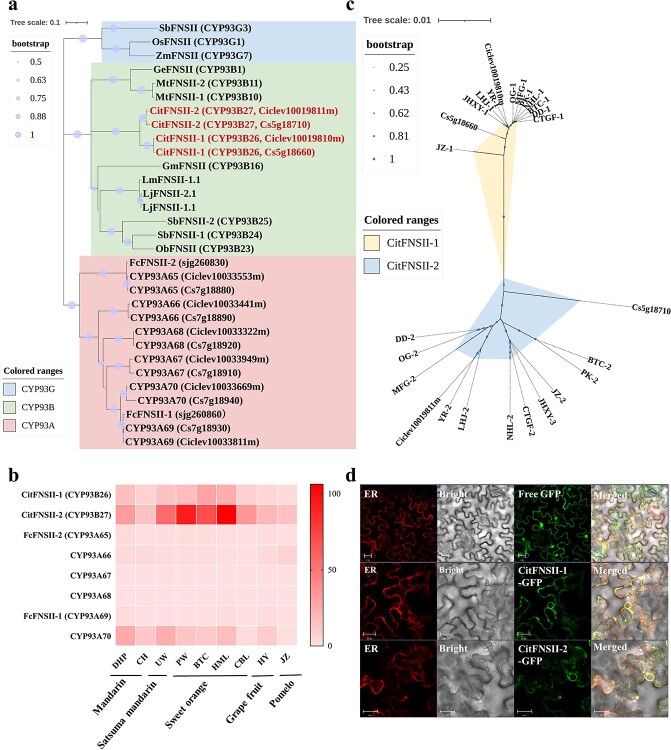
Identification of probable FNSIIs. **a** Phylogenetic analysis of citrus CYP93 members with other FNSIIs. Three main clusters (CYP93A, CYP93B, and CYP93G subfamily) are indicated in the tree, respectively with pink, pale green, and blue backgrounds. The candidate proteins are marked in red. **b** Heat map of the expression patterns of citrus CYP93 genes in various citrus varieties. The color scale on the right represents the FPKM values. The results are averages of FPKM of three biological replicates. DHP, ‘Xingyidahongpao’; CH, ‘Chachiensis’; UW, ‘Ueno wase’; PW, ‘Powell’; BTC, ‘Bingtangcheng’; HML, ‘Hamlin’, CBL, ‘Campbell’; HY, ‘Huyou’; JZ, ‘Jiangxizaoyou’. **c** Phylogenetic analysis of sequences of *CitFNSIIs* from 11 citrus germplasm. OG, ‘Ougan’; PK, ‘Ponkan’; LHJ, ‘Lihuaju’; YR, ‘Yura’; BTC, ‘Bingtangcheng’; NHL, ‘Newhall navel orange’; CTGF, ‘Cocktail grapefruit’; JHXY, ‘Juhuaxinyou’; JZ, ‘Jiangxizaoyou’; DD, ‘DaiDai sour orange’; MFG, ‘Mafenggan’. **d** Subcellular localization of CitFNSIIs. Tobacco cells were transformed with CitFNSII-1-GFP, CitFNSII-2-GFP, or free GFP in combination with an ER-mCherry marker. Scale bars = 20 μm. ER, endoplasmic reticulum.

To identify the candidate genes encoding FNSII in citrus, we constructed a phylogenetic tree to assess the evolutionary relationship between citrus CYP93 family members (including sequences from *Citrus clementina* and *C. sinensis* genome database) and FNSIIs from *O. sativa*，*S. bicolor*，*Z. mays*，*G. echinata*，*M. truncatula*，*G. max*，*Ocimum basilicum*，*S. baicalensis*，*Lonicera japonica*，*Lonicera macranthoides* and *F. crassifolia.* (Fig. 2a). The results showed that, after removing incomplete sequences, eight CYP93 proteins from citrus fell into two subfamilies, CYP93A and CYP93B. Only CYP93B26 and CYP93B27 were grouped in the same clade as other FNSIIs from CYP93B subfamily, while the homologs of the reported FcFNSIIs in kumquat fell into the CYP93A branch [38] (Fig. 2a). The expression patterns of the CYP93 genes in different cultivars were subsequently investigated basing on transcriptome analysis of mature fruits from nine representative cultivars (Fig. 2b). Of all eight CYP93 genes, only CYP93B26 and CYP93B27 were expressed at relatively high levels in all analysed cultivars, especially in mandarins and sweet oranges, which accumulate flavones in large quantities [1, 6]. In contrast, the *CYP93A65* and *CYP93A69* that were homologous to the FcFNSIIs in kumquat were barely expressed in all the nine citrus cultivars. Thus, we hypothesized that the members responsible for flavone synthesis in *Citrus* might be different from those in *Fortunella*, and were more likely to be the two members of 93B, which are termed as CitFNSII-1 (CYP93B26) and CitFNSII-2 (CYP93B27), respectively. *CitFNSII-2* shared 95.63% nucleotide identity with *CitFNSII-1*, and both of them were located on scaffold 3 (Genome of *Citrus clementina*) within 100 kb gene distance. According to the collinearity and gene duplication analysis in our previous report [39], these two members should belong to duplicated genes. In addition, the phylogenetic analysis showed that CitFNSII-1 and CitFNSII-2 were also clustered with GeFNSII, MtFNSII-1, and MtFNSII-2, which are reported to be involved in 2-hydroxyflavanone biosynthesis [19, 22], indicating that CitFNSII-1 and CitFNSII-2 might have a similar function in citrus (Fig. 2a).

To investigate the sequence variation of two *FNSII* gene copies in different citrus germplasms, we amplified the coding regions of *CitFNSII-1* and *CitFNSII-2* from 11 representative citrus germplasms (including primitive germplasms and current cultivars) and performed phylogenetic analysis. Because these two genes possess highly similar nucleotide sequences, we initially amplified them with the same pair of primers. However, the sequence of *CitFNSII-1* was not obtained in sweet orange and satsuma mandarin, so we changed a more specific primer to re-amplify it (Table S1, see online supplementary material). The phylogenetic analysis of re-sequencing results showed that *CitFNSII-1* and *CitFNSII-2* could be divided into two distinct branches, and each gene was highly conservative in the process of germplasm evolution (Fig. 2c). Amino acid sequence alignment revealed that both CitFNSII-1 and CitFNSII-2 proteins harbored the typical sequence signatures of the CYP450 superfamily, such as an oxygen-binding pocket, a proline-hinge region, and a heme-binding motif (Fig. S1, see online supplementary material).

The CYP450s were reported to be located on endoplasmic reticulum (ER) [23]. We investigated the subcellular localization of CitFNSII-1 and CitFNSII-2 by overexpressing the C-terminally tagged GFP fusion proteins in the leaves of *Nicotiana benthamiana*. The ER structure in the tobacco cells were marked with an ER-mCherry protein. Compared with the control tobacco leaf epidermal cells (with Free GFP) whose nucleus and cytoplasmic space were both with observed fluorescent signals, the fluorescent signals of the fusion proteins (with CitFNSII-1-GFP or CitFNSII-2-GFP) were network-like (Fig. 2d), and most of the signals merged with the fluorescence signals of the ER-marker. These indicted that the CitFNSII-1 and CitFNSII-2 were located in ER as other CYP450s.

### *In vivo* yeast expression and enzyme assays of recombinant CitFNSII proteins

**Figure 3:**
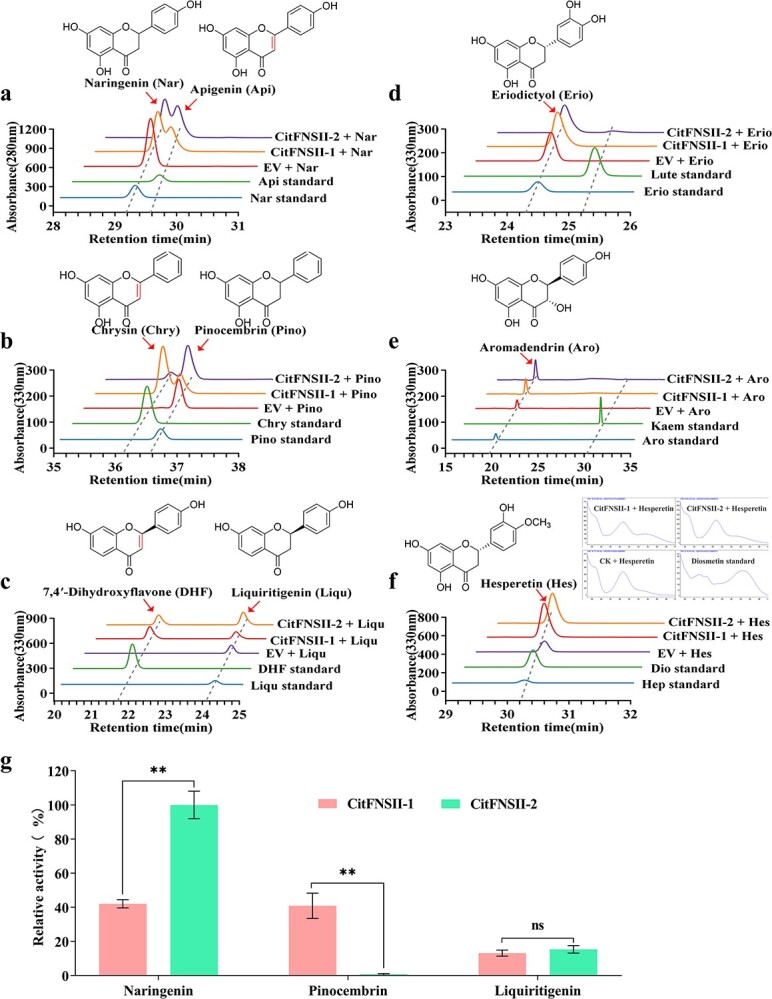
*In vivo* yeast enzyme-activity assays of CitFNSII-1 and CitFNSII-2. **a**–**f** HPLC analyses of products derived from various substrates incubated with recombinant CitFNSII-1 and CitFNSII-2 in yeast. The assays were conducted with six substrates: (**a**), Nar, naringenin; (**b**), Pino, pinocembrin; (**c**), Liqu, liquiritigenin; (**d**), Erio, eriodictyol; (**e**), Aro, aromadendrin; (**f**), Hes, hesperetin. EV, yeast transformed with empty pYES2 NT/C vector; CitFNSII-1, yeast transformed with recombinant CitFNSII-1 vector; CitFNSII-2, yeast transformed with recombinant CitFNSII-2 vector. **g** Relative activity of CitFNSII-1 and CitFNSII-2 in catalyzing naringenin, pinocembrin, and liquiritigenin to generate corresponding flavones. Error bars represent SE from three technical repeats. Statistical analysis: Student's *t*-test. ^**^P < 0.05.

The transformed yeast cells expressing CitFNSII-1 and CitFNSII-2 were able to convert naringenin (with 5-OH, 7-OH, and 4${}^{{\prime}}$-OH groups), pinocembrin (with 5-OH and 7-OH groups), and liquiritigenin (with 7-OH and 4′-OH groups), to their corresponding flavones: apigenin, chrysin, and 7, 4′-dihydroxyflavone (DHF), respectively (Fig. 3a–c; Fig. S2, see online supplementary material). When using eriodictyol (with 5-OH, 7-OH, 3′-OH, and 4′-OH groups) as substrate, an inconspicuous small peak was detected in the transformed yeast cells expressing CitFNSII-2 (Fig. 3d). LC–MS/MS analysis confirmed that the product was luteolin (Fig. S2, see online supplementary material). When using aromadendrin (3-OH, 5-OH, 7-OH, and 4′-OCH_3_ groups) and hesperetin (5-OH, 7-OH, 3′-OH, and 4′-OCH_3_ groups) as substrates, no new products were observed in both transformed yeast cells expressing CitFNSII-1 and CitFNSII-2 (Fig. 3e–f). Thus, we speculated that these two CitFNSII proteins possessed extremely low or no catalytic activity for substrates containing a 3′-OH or a 3-OH. These results indicated that in the process of citrus flavones synthesis, the catalytic reaction of CitFNSII-1 and CitFNSII-2 might occur before the generation of 3-OH or 3′-OH. In addition, we found that when using pinocembrin as the substrate, the products of CitFNSIIs enzyme assays showed a stable unknown peak, which were confirmed to be 2-OH pinocembrin based on the molecular weight and the ion fragments detected by the LC–MS (Fig. S3, see online supplementary material). This indicated these two CitFNSIIs might also possess the ability to function as 2-hydroxyflavanone enzyme like their homologues GeFNSII, MtFNSII-1, and MtFNSII-2 [19, 22].

The determination of the enzymatic kinetics of the CYP450s family members is a difficult task because of the limitation in the concentration of membrane proteins and the purification method. Therefore, we applied the yeast heterologous expression method to analyse their relative catalytic activities for different substrates, so as to preliminarily explore their substrate preferences. The CitFNSII-2 showed higher catalytic activity than CitFNSII-1 when taking naringenin as substrate, while lower catalytic activity than CitFNSII-1 when taking pinocembrin as substrate. When taking liquiritigenin as a substrate, the two CitFNSIIs showed an equivalent catalytic activity (Fig. 3g). CitFNSII-1 exhibited a similar catalytic activity to both naringenin and pinocembrin, while CitFNSII-2 only presented an extremely high catalytic activity to naringenin, the proposed precursor of flavones synthesis in citrus (Fig. 3g). This indicated that CitFNSII-1 and CitFNSII-2 possess different substrate preferences, and might be responsible for the synthesis of different flavones in citrus.

To further figure out the structural basis for the differences in relative catalytic activities, the naringenin binding with CitFNSII-1 and CitFNSII-2 were modeled respectively (Fig. S4, see online supplementary material). *In silico* docking analysis showed that naringenin bound in a similar orientation at the active sites of both enzymes, but occupied a tilted position in CitFNSII-2. In the binding model of naringenin and CitFNSII-1, the residues such as Leu105, Thr106, Ser109, Ile368, Ser369, Phe431 were predicted to be a part of the ligand-binding pocket, and pushed the substrate toward the active site. While in CitFNSII-2, residue Leu368 formed a pi-sigma interaction with the A ring of naringenin, which caused a tilted conformation and a closer distance between C2 of naringenin and the heme plane. Meanwhile, a hydrophobic pocket composed of Phe111 and Leu432, formed pi-pi T-shaped and pi-sigma interaction with the substrate and stabilized such tilted conformation, which was absent in the binding model of CitFNSII-1. As a result, the C2 of naringenin was within 19.2 Å of the heme at the active site in CitFNSII-1, and the relative proximity (17.5 Å) in that of CitFNSII-2 might drive its higher catalytic efficiency to produce apigenin (Fig. S4, see online supplementary material). According to homology modeling results, the different residues at 368 and 369 might be responsible for the conformational differences between the two proteins (Fig. S4, see online supplementary material).

### Expression profiles of *CitFNSII* genes in different citrus cultivars and developmental stages

**Figure 4:**
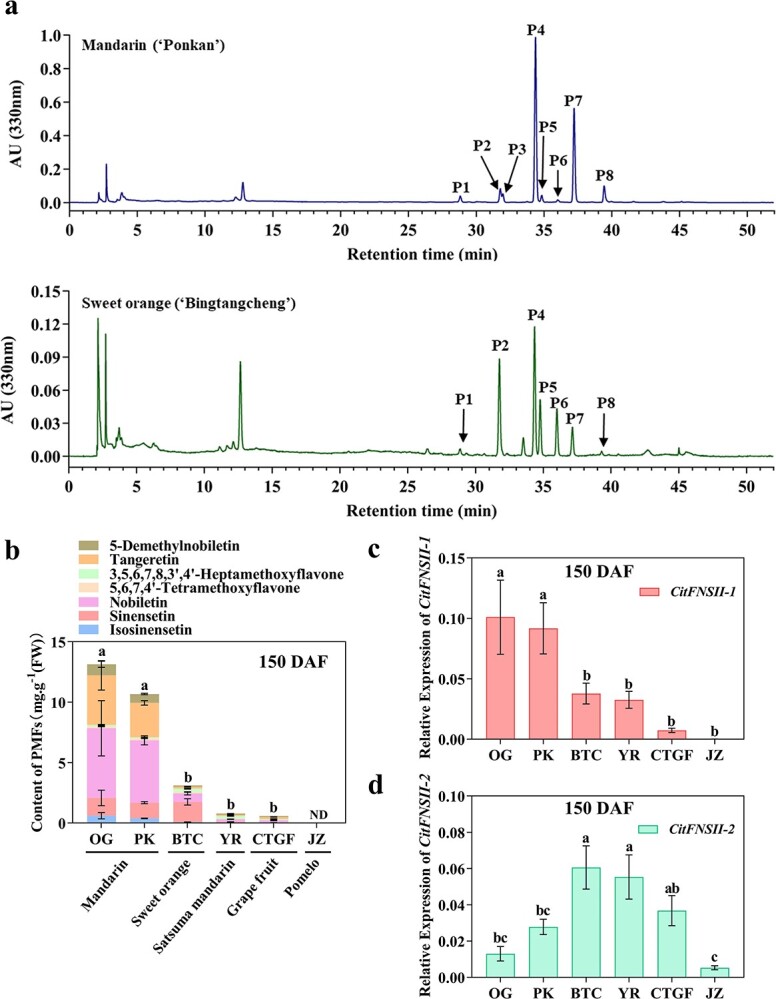
Comparison of PMF contents and *CitFNSIIs* transcript levels in six citrus cultivars. **a** Representative HPLC chromatograms of PMFs in mandarin and sweet orange at 330 nm. Peak 1 (P1), isosinensetin; Peak 2 (P2), sinensetin; Peak 3 (P3), 5,7,8,4′-tetramethoxyflavone; Peak 4 (P4), nobiletin; Peak 5 (P5), 5,6,7,4′-tetramethoxyflavone; Peak 6 (P6), 3,5,6,7,8,3′,4′-heptamethoxyflavone; Peak 7 (P7), tangeretin; Peak 8 (P8), 5-demethylnobiletin. **b** PMF contents in citrus flavedo of six cultivars at 150 days after flowering (DAF). **c**–**d** Relative expression of *CitFNSII-1* (**c**) and *CitFNSII-2* (**d**) in citrus flavedo of six cultivars at 150 DAF. PK, ‘Ponkan’; YR, ‘Yura’; OG, ‘Ougan’; BTC, ‘Bingtangcheng’; CTGF, ‘Cocktail grapefruit’; JZ, ‘Jiangxizaoyou’. Error bars indicated SE from three biological repetitions. The lowercase letters indicated the significant differences among different cultivars (*P* < 0.05), one-way ANOVA with Duncan’s multiple range test.

Six cultivars fruits of 150 DAF, including mandarin (‘Ougan’, OG; ‘Ponkan’, PK), sweet orange (‘Bingtangcheng’, BTC), satsuma mandarin (‘Yura’, YR), grapefruit (‘Cocktail grapefruit’, CTGF), and pomelo (‘Jiangxizaoyou’, JZ) were chosen to analyse the PMFs contents, as well as the transcript levels of both *CitFNSII-1* and *CitFNSII-2* in the flavedo. Abundant PMFs were detected in both mandarin fruit and sweet orange (Fig. 4a; Fig. S5, see online supplementary material). The mandarin fruit (OG and PK) accumulated the richest PMFs, following sweet orange (BTC) (Fig. 4b). In addition, nobiletin and tangeretin were the main PMFs in mandarin, while sinensetin was the main PMF in sweet orange. Satsuma mandarin (YR) and grapefruit (CTGF) accumulated fewer PMFs, and no PMFs was detected in pomelo (JZ) (Fig. 4b). *CitFNSII-1* showed a high expression in mandarin (OG and PK), while *CitFNSII-2* is highly expressed in sweet orange (BTC) and satsuma mandarin (YR) (Fig. 4c and d). Both *CitFNSII-1* and *CitFNSII-2* showed extremely low transcript levels in pomelo (JZ) (Fig. 4c and d). The expression levels of these two *CitFNSII*s was positively related to the PMFs accumulation level, and the correlation between *CitFNSII-1* expression levels with PMFs contents in different cultivars was higher (Fig. 4b and c).

**Figure 5:**
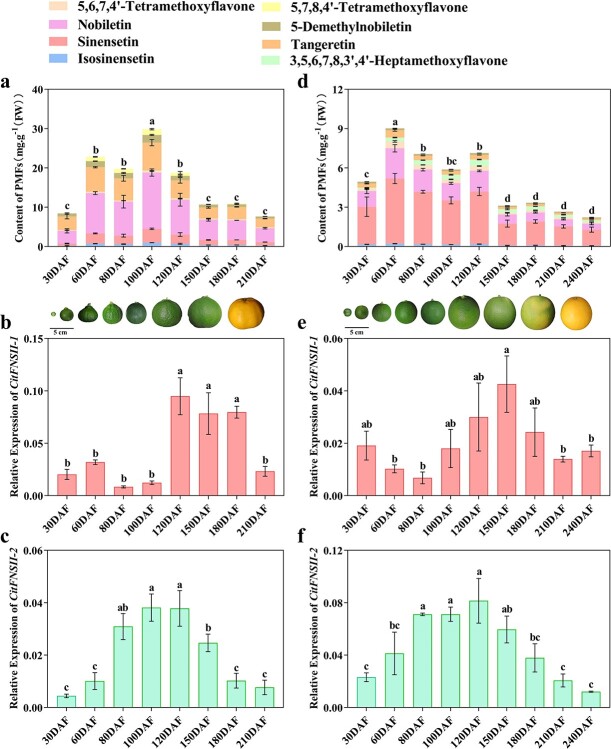
Dynamic changes of PMF contents during PK and BTC fruit development. **a** PMFs contents in fruit flavedo during PK fruit development. **b** Transcript levels of *CitFNSII-1* in flavedo during PK developmental stages. **c** Transcript levels of *CitFNSII-2* in flavedo during PK developmental stages. **d** PMFs contents in fruit flavedo during BTC fruit development. **e** Transcript levels of *CitFNSII-1* in flavedo during BTC developmental stages. **f** Transcript levels of *CitFNSII-2* in flavedo during BTC developmental stages. Error bars indicated SE from three biological repetitions. The lowercase letters indicated the significant differences among different developmental stages (*P* < 0.05), one-way ANOVA with Duncan’s multiple range test. DAF, days after flowering; FW, fresh weight; Bars = 5 cm.

Subsequently, we chose PK and BTC fruits to analyse the transcript levels of *CitFNSII*s and the changes of PMFs contents during the fruit development process. As shown in Fig. 5, PK and BTC exhibited similar pattern of PMF accumulations. The fruit of these two cultivars from 60 DAF to 120 DAF accumulated relatively higher levels of PMFs in flavedo, which dropped sharply at 150 DAF, and then remained a relative stable level. Correspondingly, the expression pattern of *CitFNSII-1* and *CitFNSII-2* presented a respective similarity in PK and BTC. The increment of *CitFNSII-1* transcript levels in both PK and BTC slightly lagged behind the PMFs accumulation, which peaked during the 120–180 DAF (Fig. 5b and e). In comparison, the transcript levels of *CitFNSII-2* increased initially and peaked at around 80–120 DAF, with the change patterns in line with the PMFs accumulation patterns (Fig. 5c and f). This indicated that in the developmental process of PK and BTC fruits, the expression of *CitFNSII-2* was more relative to PMFs accumulations.

### Homologous function validation of CitFNSIIs in citrus

**Figure 6:**
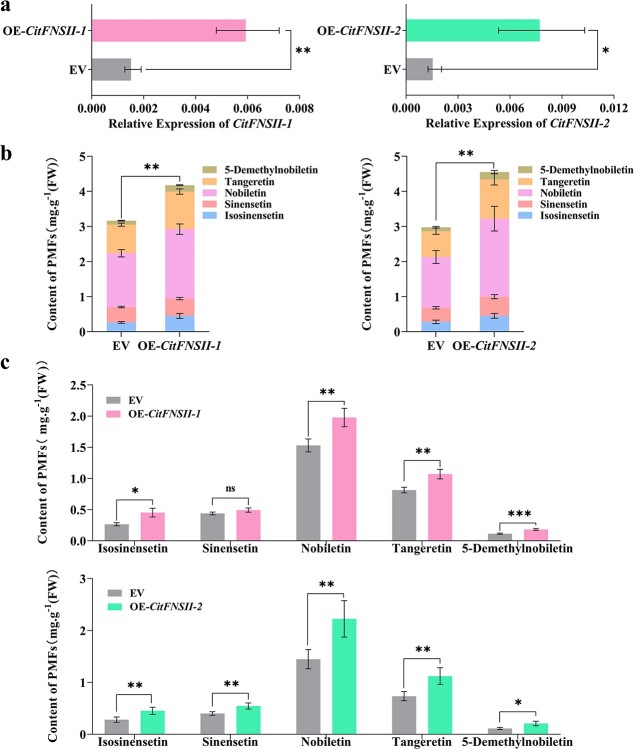
The effects of transient overexpression of *CitFNSII-1* and *CitFNSII-2* on PMF contents in mature PK fruit peels. **a** Expression of *CitFNSIIs* in PK peels at fourth day after injection. **b** The content of total PMFs after *CitFNSIIs* were overexpressed. **c** The contents of single PMF after *CitFNSIIs* were overexpressed. The content of PMFs is calculated as mg g ^−1^ FW (fresh weight). Error bars represented SE from nine biological repetitions. Statistics analysis: Student’s paired *t*-test. ^*^P < 0.05, ^**^P < 0.01; ^***^P < 0.001.

To explore the *in vivo* functions of *CitFNSIIs* in the biosynthesis of flavones, the mature fruits of mandarin PK were used to conduct the homologous overexpression experiments. Our present studies have shown that the PMFs as well as the expression levels of the *CitFNSII-1* and *CitFNSII-2* in mature fruits were low and showed no real difference. After infiltration with *Agrobacterium* strain *CitFNSIIs-pBI121*, the gene transcript levels of both *CitFNSII-1* and *CitFNSII-2* in PK peel improved significantly, compared with the control, which were infiltrated with empty vector (Fig. 6a). Transient overexpression of *CitFNSIIs* in PK peel, either *CitFNSII-1* or *CitFNSII-2,* obviously increased the accumulation of total PMFs content: transient overexpression of *CitFNSII-1* or *CitFNSII-2* increased the accumulation of total PMFs content by 32% and 53%, respectively (Fig. 6b); the contents of single PMF were all presented with different degrees of increase (Fig. 6c).

**Figure 7:**
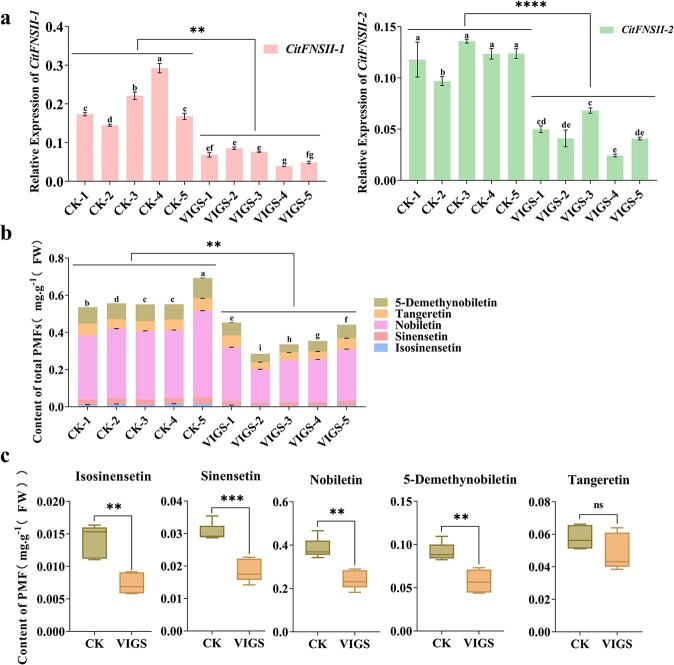
Virus-induced *CitFNSII*s silencing in PK seedlings. **a** Relative expression of *CitFNSII-1* and *CitFNSII-2* in virus-induced *CitFNSII*s silencing PK seedlings. **b** The effect of virus-induced *CitFNSIIs* silencing on the contents of total PMFs. Error bars represented SE from three technical repetitions in (**a**) and (**b**); the lowercase letters indicated the significant differences among different single lines (*P* < 0.05), one-way ANOVA with Duncan’s multiple range test. **c** Measurements of single PMF after silenced *CitFNSIIs* in PK seedlings. CK, plants of empty TRV2 vector; VIGS, plants of virus-induced *CitFNSII*s silencing. Error bars represented SE from five biological repetitions in (**c**). Statistical difference between CK and VIGS groups was examined by Student’s *t*-test. ^**^P < 0.01; ^***^P < 0.001; ^****^P < 0.0001.

The results of virus-induced gene silencing (VIGS) further confirmed the functions of *CitFNSII*s. A 297-bp coding region highly conserved (99%) of CitFNSII-1 and CitFNSII-2 was transformed into PK germinated seedlings by TRV vector to down-regulate the expressions of *CitFNSII-1* and *CitFNSII-2* simultaneously. The transcript levels of *CitFNSII-1* and *CitFNSII-2* in 5 VIGS-positive lines, respectively, presented a 68% and 62% reduction compared with the control plants that were transformed with empty vector TRV2 (Fig. 7a). Correspondingly, the total contents of PMFs in the positive lines exhibited a 35% decrease compared with the control plants (Fig. 7b). Single PMF such as isosinensetin, sinensetin, nobiletin, as well as 5-demethynobiletin all observed a notable reduction (Fig. 7c). The above results demonstrated that both *CitFNSII-1* and *CitFNSII-2* were involved in the biosynthesis of flavones in citrus, and thus promoted the PMFs production.

### MeSA treatment affected the accumulation of flavones and the expression of *CitFNSII-2*

**Figure 8:**
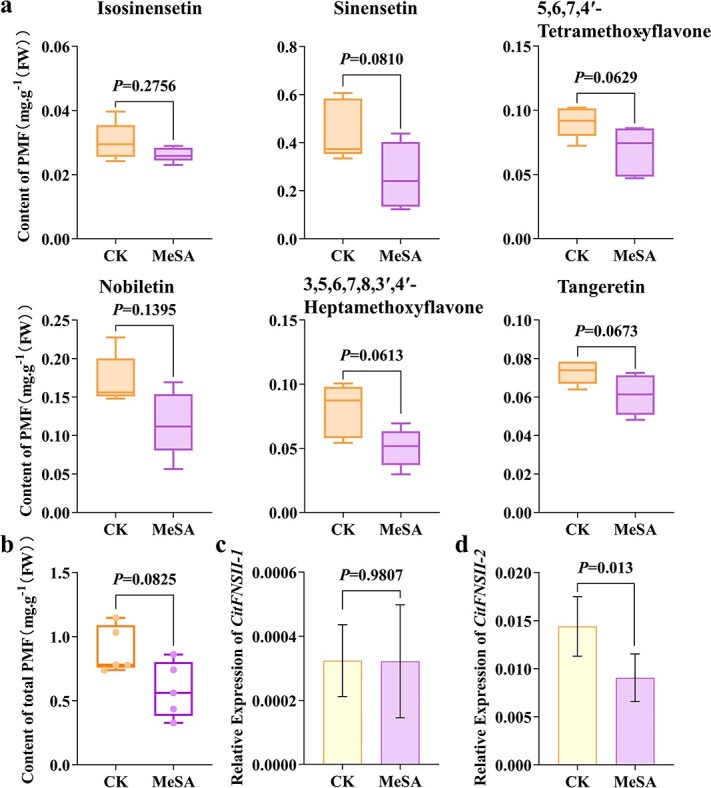
PMF contents and *CitFNSIIs* transcript levels of BTC after MeSA treatment. **a** Changes of single PMF in the peel of BTC fruit after MeSA treatment. **b** Changes of total PMFs in the peel of BTC fruit after MeSA treatment. **c** Expression levels of *CitFNSII-1* after MeSA treatment. **d** Expression levels of *CitFNSII-2* after MeSA treatment. Error bars represented SE from five biological repetitions. Statistics analysis: Student’s paired *t*-test.

MeSA has been reported to influence the accumulations of flavonoids, but its effect on the branch of flavone synthesis is rarely studied. As the results show in the present study, 1 mmol/L MeSA treatment decreased the content of PMFs in the peel of BTC fruit. The sinensetin, tangeretin, 5,6,7,4′-tetramethoxyflavone, and 3,5,6,7,8,3′,4′-heptamethoxyflavone showed a more significant decrease (Fig. 8a). The total PMFs contents in the treated peel decreased by 35% compared with the control (Fig. 8b), indicating that treatment with MeSA might suppress PMFs production. A quantitative RT-PCR analysis showed that the transcript levels of *CitFNSII-2* in treatment groups significantly decreased by 38%, while the transcript levels of *CitFNSII-1* showed no significant change (Fig. 8c and d). This finding demonstrated that in BTC fruit, CitFNSII-2 rather than CitFNSII-1 was more sensitive to MeSA treatment and participated to mediate the changes of PMFs contents under the influence of exogenous MeSA.

## Discussion

The synthesis pathway of citrus PMFs originated from the phenylpropane pathway. Previous studies have identified CitCHIL1 and CitOMTs from citrus and verified their catalytic activities and functions [5, 40], while FNS, the critical intermediate enzyme has not been characterized in citrus yet. Here, we cloned two CitFNSIIs from citrus and clarified their catalytic function in yeast as well as *in planta*. Moreover, we found that these two genes had different expression patterns in different citrus cultivars as well as developmental stages.

### CitFNSII-1 and CitFNSII-2 were involved in citrus flavone biosynthesis

The results of *in vivo* yeast enzyme assays showed that both FNSII proteins could catalyze flavanones without a 3′-OH or a 3-OH to produce flavones (Fig. 3). However, these two CitFNSIIs exerted differences in catalytic activity for different substrates. Apparently, they both exhibited higher catalytic activity to naringenin, which acted as the primary precursor in flavonoids biosynthesis pathway in citrus (Fig. 3g). Although these two CitFNSII proteins possessed very similar amino acid sequences, the relative catalytic activity of CitFNSII-2 to naringenin exceeded that of CitFNSII-1. According to the molecular docking results, when naringenin bound to CitFNSII-2, its C2 site was closer to the heme at the active site, which might cause the higher substrate affinity of CitFNSII-2 to naringenin (Fig. S4, see online supplementary material). This phenomenon is similar to the reason that causes the substrate-specificity of CYP82D1.1 and CYP82D2 in *S. baicalensis* [24]. We speculated that the difference in conformation of CitFNSII-2 and CitFNSII-1 binding naringenin might be related to the substitutions of the two residues at 368 and 369, which were Ile368 and Ser369 in CitFNSII-1 and Leu368 and Asn369 in CitFNSII-2, respectively (Fig. S1 and S4, see online supplementary material) [41]. Additionally, CitFNSII-1 also presented a superior catalytic activity to pinocembrin, while CitFNSII-2 could hardly catalyze pinocembrin. We also observed that when using pinocembrin as substrate, two CitFNSIIs exhibited the F2H activity to produce 2-OH pinocembrin (Fig. S3, see online supplementary material), like their homologous proteins GeFNSII, MtFNSII-1, and MtFNSII-2 (CYP93B1, CYP93B10, and CYP93B11) [19, 22]. However, we did not observe similar by-products in the products of enzyme reactions using naringenin or liquiritigenin as substrates, and thus we cannot determine whether CitFNSIIs can catalyze these two flavanones to generate the corresponding 2-OH modification products. Because some type II FNSs can produce 2-hydroxyflavanones as intermediates in the process of catalyzing the formation of the double bond at 2–3 site of the flavones [25], we speculated that 2-hydroxylased intermediates for naringenin and liquiritigenin might be too unstable to detect due to the limitations of the reaction system and detection system. CitFNSII-1 and CitFNSII-2 produced no or extremely low luteolin, while luteolin derivatives exist in citrus [42, 43]. Hence, we inferred that there might be other undetected FNSI or FNS-like in citrus to perform this function. For example, a tomato polyphenol oxidase (SlPPO F) was reported to function as a FNS-like, catalyzing the conversion of eriodictyol to luteolin [10]. The homologous *in planta* functional verification of CitFNSII-1 and CitFNSII-2 demonstrated that in these two enzymes there existed no significant difference in participation in PMF biosynthesis, implying that they might perform congenerous functions, thus promoting flavone accumulation in citrus.

### Expression patterns of *CitFNSII-1* and *CitFNSII-2* vary among developmental stages and varieties

Phylogenetic analysis of two *CitFNSIIs* genes from 11 representative citrus germplasms demonstrated both CitFNSII-1 and CitFNSII-2 were highly conservative in the process of variety evolution (Fig. 2c). Hence, we speculated that these two genes were generated from gene duplication before germplasm domestication. Previous studies have shown that PMFs initially increased and then decreased during the fruit development of citrus [44]. The mandarin fruit accumulated the most abundant PMFs in the flavedo, followed by sweet oranges, while the pomelo fruit hardly accumulated PMFs [6]. Satsuma mandarin fruit accumulated much higher levels of flavanones and extremely low levels of PMFs compared with PK [45]. Our results were consistent with the previous reports (Fig. 4). In addition, we also found that, as duplicated genes, the expression pattern of *CitFNSII-1* was highly correlated with PMF contents in different cultivars while the expression pattern of *CitFNSII-2* was more correlated with PMF accumulations in fruit development stages. The transcriptome sequencing showed the different expression patterns between these two duplicated genes in BTC development stage (Fig. S6, see online supplementary material). Hence, we analysed the gene structure of *CitFNSIIs* and observed that the UTR region and promoter region were totally different in these two genes, so we speculated that the main reason of different expression patterns of these two duplicated genes might be distinct regulatory factors and mechanisms.

### Reduced PMFs content by MeSA is associated with decreased *CitFNSII-2* expression levels

Phytohormone MeSA have been testified to regulate the accumulation of flavonoids [37]. We observed the decrease of PMFs accumulation in the MeSA treatment groups (Fig. 8a and b). Subsequently, we further investigated the expression levels of *CitFNSII*s, and found the expression levels of *CitFNSII-2* decreased significantly, compared with the control, while the expression levels of *CitFNSII-1* showed no change (Fig. 8c and d). This result indicated that compared with CitFNSII-1, the CitFNSII-2 was more sensitive to MeSA, and these two genes might have completely different regulation mechanism. We figured that this might attribute to the lower expression level of *CitFNSII-1* in BTC flavedo (Fig. S6, see online supplementary material), and the decrease of PMFs content was directly relative to the decrease of *CitFNSII-2* expression level, which further emphasized the correlation between *CitFNSII-2* expression and the accumulation of PMFs. In *S. baicalensis*, MeJA could enhance the root-specific flavones and transcript level of *SbFNSII-2*, while having no effect on *SbFNSII-1*, and the SbFNSII-2 was more highly expressed in the roots and proved to play a more important role in the synthesis of root-specific flavones in *S. baicalensi* [24]. In *Bidens pilosa* leaves, MeSA and MeJA treatments might induce similar metabolic pathways [46]. Besides, in tea (*C. sinensis*) leaves, a moderate concentration of MeSA (1 mmol/L) was proven to promote the biosynthesis of flavonoids by stimulating the phenylpropanoid pathway. The study of tea also showed a time-dependent accumulation of flavonoids after treatment with 1 mmol/L MeSA. They found flavonoid concentration increased at first and then decreased, reaching the maximum level at 2 days post-treatment, and decreasing to a lower level than that of control after 6 days [37], which was consistent with our current results. In the leaves of wheat, SA treatment up-regulated the genes related to the flavonol metabolism, such as *F3H* and *DFR*, and increased the flavonol content [47]. We suspected that this might be due to the biosynthesis of flavones and flavonols as competitive pathways, which were catalyzed by FNS and FLS, respectively. However, the mechanism that MeSA treatment led to the decrease of PMFs at 6 days needs to be explored further.

### Conclusion

In summary, we identified two CitFNSIIs (CitFNSII-1 and CitFNSII-1) and verified their functions in this study. We also demonstrated their conservative property in the evolution of varieties; and their differences of expression patterns both in common cultivated varieties and various development stages. These findings will contribute to the refinement of citrus flavonoid synthesis pathways and further investigation of flavonoid metabolic pathways in plants.

## Materials and methods

### Plant materials and treatment

‘Bingtangcheng’ (*C. sinensis* (L.) Osbeck), ‘Ponkan’ (*C. reticulata* Blanco cv. Ponkan), ‘Ougan’ (*C. reticulata* cv. Suavissima), ‘Yura’ (*Citrus unshiu* Marc.), Cocktail grapefruit (*Citrus paradisi* Macf.) were cultivated in Quzhou, Zhejiang province. ‘Jiangxizaoyou’ (*Citrus grandis* (L.) Osbeck) were cultivated in Beibei, Chongqing. The fruits were gathered at 30, 60, 80, 100, 120, 150, 180, 210, and 240 days after full flowering period (DAF), respectively. The flavedo was scraped from the fruits, instantly frozen with liquid nitrogen, and stored at −80°C until use. For each time point, three biological replicates were set up, with four fruits in each replicate.

‘Bingtangcheng’ fruits harvested at 180 DAF were treated with 1 mmol/L MeSA. For each fruit, 5 mL of 1 mmol/L MeSA dissolved in distilled water (as treatment) and distilled water (as control) were injected into the peel on opposite sides of the equatorial plane, respectively. The diameter of injection area is about 4 cm. After injection, the samples were laid in room temperature and in darkness. The injection parts of fruit peels were then sampled at 6 days after treatment. Five biological replicates were done with one single fruit as a biological replicate.

### Chemicals

Naringenin, apigenin, eriodictyol, pinocembrin, liquiritigenin, and other standards were bought from Yuanye Bio-Technology (Shanghai, China). 2-OH pinocembrin was bought from Chengdu SinoStandards Bio-Tech (Chengdu, China). Methanol and acetonitrile for HPLC were bought from Sigma-Aldrich (St. Louis, MO, USA). MeSA solution used for treatment was bought from Sangon Biotech (Shanghai, China).

### RNA extraction, RNA-seq, and RT-PCR

Total RNA of samples was extracted according to a previous protocol [48], and 1 μg of total RNA was processed via a PrimeScript™ RT Reagent Kit (Takara, Dalian, China) to eliminate genomic DNA (gDNA) contamination and synthesize the cDNA strand. Gene transcript levels detected in RNA-seq were estimated by FPKM (fragments per kilobase of exon per million fragments mapped). Real-time quantitative–PCR (RT–PCR) was carried out as mentioned before [5], to verify the expression levels of *CitFNSII-1* and *CitFNSII-2*. Each quantitative PCR was performed for three individual times independently. Gene-specific primers used for RT-PCR are listed in Table S1 (see online supplementary material), and the specificity of primers was tested and confirmed by product resequencing. Citrus *β*-actin served as a house-keeping gene for normalization [49] and the relative abundance of gene transcripts was measured with 2^∆Ct^.

### Phylogenetic analysis and sequence alignment

According to the number determined by the naming committee, the sequence of citrus CYP93 members were blasted from the *C. clementina* genome database and the *C. sinensis* genome database. The deduced amino acid sequences of citrus CYP93 family members then were aligned with already-identified FNSIIs in different species (Table S2, see online supplementary material). The MEGA X software was used to construct the phylogenetic tree in Fig. 2 [50], using N-J (Neighbor-Joining) method and testing with 1000 bootstrap replicates. Beautification and annotation of the phylogenetic tree was performed employing the online iTOL (https://itol.embl.de/). The results of sequence alignment were visualized through ESPript 3.0 (https://espript.ibcp.fr/). The nucleotide sequences from various citrus germplasms were cloned using different primers listed in Table S1 (see online supplementary material) and then a resequencing performed. The information of FNSIIs used to perform sequence alignment and construct the phylogenetic tree was listed in Table S2 (see online supplementary material). An unrooted tree of these nucleotide sequences was constructed as described above.

### Homology modeling and molecular docking

In order to establish suitable protein templates for homology modeling, searches were conducted with the amino acid sequence of CitFNSII-1 and CitFNSII-2, respectively, at the website of SWISS-MODEL (https://swissmodel.expasy.org/). The protein 8E83_A (8e83.1.A) was chosen as the template for its high sequence similarity with CitFNSIIs. The 3D homology models of CitFNSII-1 and CitFNSII-2 were generated in the SWISS-MODEL sever, respectively, and the 3D structures of heme and naringenin were downloaded from the PubChem database (https://pubchem.ncbi.nlm.nih.gov/). Molecular docking was performed according to a previous report [51] by using the AutoDock Vina [52].

### Flavonoids extraction and measurement

The extraction of total flavonoids was conducted according to previous study [5] with minor modification. A Waters HPLC system equipped with a 2695 quaternary pump and a 2996 diode array detector (Waters Corp., Milford, MA, USA) was used in the chromatographic experiments. A Sunfire C18 ODS column (4.6 × 250 mm, 5 μm, Waters Corp.) was used to separate the compounds at room temperature with a flow rate of 1 mL min^−1^. The mobile phases were water (A) and acetonitrile (B) with a linear gradient program as follows, 0/80, 5/80, 10/73, 15/73, 25/60, 35/40, 40/20, 42/0, 45/80, and 50/80 (min/A%) [40]. The compounds were monitored under 280 nm and 330 nm, and quantitated according to the standard curves of the authentic standards [40]. The enzymatic reaction products were detected using an Agilent 1290 Infinity HPLC system (quaternary pump, a DAD detector) and the compounds were identified combining ion fragments, retention time, and characteristic spectrum.

### LC–MS/MS analysis

A Waters UPLC system (Waters Corp., Milford, MA, USA) and an ACQUITY UPLC Agilent ZORBAX-SB C18 column (4.6 × 100 mm, 1.8 μm) was used in the chromatographic experiments of LC–MS. Other LC conditions were referred to the method for flavonoid measurement above. An AB TripleTOF 5600plus System (AB SCIEX, Framingham, MA, USA) was used to perform mass spectrum analysis. The scanning range (m/z) is 100–1500 Da; The pressures of atomized gas (GS1), atomized gas (GSI2) and curtain gas (CUR) were 55 psi, 55 psi, and 35 psi, respectively. MS spectra were obtained in negative (or positive) ion mode (ESI). Negative ion scanning mode: ion source temperature (TEM) 550°C, voltage (IS) −4500 V; Positive ion scanning mode: ion source temperature (TEM) 600°C, voltage (IS) 5500 V; First-stage scan mode: 100 V declustering voltage (DP), 10 V focusing voltage (CE); Secondary scanning mode: relying on the product ion scanning mode to acquire secondary mass spectrometry information, the collision energy is 40 ± 20 (positive), −40 ± 20 (negative). Automated Calibration Delivery System is used to correct the mass axis.

### Yeast expression of recombinant proteins

Yeast expression and *in vivo* yeast assays were carried out referring to prior methods [41], with several modifications. The *CitFNSII-1* and *CitFNSII-2* were isolated referring to the *C. clementina* genome. The coding sequence (CDS) of *CitFNSII-1* and *CitFNSII-2* were cloned using the primers listed in Table S1 (see online supplementary material) and inserted into a pYES2 NT/C vector. The recombinant vectors, as well as an empty pYES2 NT/C vector, were transformed into *Saccharomyces cerevisiae* strain INVSc1, respectively, using Quick Easy Yeast Transformation Mix Kit (Takara, Dalian). The transformants were grown overnight in 10 mL liquid Synthetic Dextrose Minimal Medium without Uracil (SD-Ura) with glucose (20 g L^−1^) at 30°C. Cells were centrifuged and resuspended in 10 mL SD-Ura liquid medium containing galactose (20 g L^−1^), then diluted to an optical density (at 600 nm; OD_600_) of 0.75 to induce expression of the recombinant proteins. Twelve hours later, the substrate naringenin, eriodictyol, pinocembrin, or liquiritigenin was added, respectively, into the medium to a concentration of 20 μmol/L for enzymatic reaction. After 12 h incubation, ethyl acetate was added to the yeast liquid to terminate the reactions. The extraction phase of ethyl acetate was dried and dissolved in a total of 200 μL of methanol for HPLC or LC–MS analysis.

For the assay of relative catalytic activities, every reaction system contained 3 mL yeast culture and the final concentration of substrate was 50 μmol/L. The concentration of yeast solution before induction was strictly controlled at OD_600_ of 0.75. After 12 h induction expression, the yeast solution was divided into four tubes and each tube contained 3 mL yeast culture. One tube of 3 mL yeast solution was used to extract the total yeast protein according to a previous method with some modification [53], and the contents of the total protein were determined via the Modified BCA Protein Assay Kit (Sangon Biotech, Shanghai, China). The substrate was added into another three tubes of yeast solution for the reaction and as three biological replicates. Finally, the product content was quantified by standard curves and the catalytic activities of different enzymes were calculated according to the specific activity: the enzyme activity of recombinant proteins = the content of the product in a reaction system (μmol) / the content of yeast total proteins in the reaction system (μg), and the relative catalytic activities of proteins for different substrates were calculated by defining the highest enzyme activity as 100%.

### Subcellular localization

The full-length CDS without termination codon of *CitFNSIIs* were respectively inserted into the pCAMBIA1300-eGFP vector to construct a GFP-tagged fusion protein, and the recombinants were transferred into *Agrobacterium tumefaciens* strain GV3101 through electroporation. The transformants carrying recombinant vectors or the empty vector were primarily grown in liquid LB broth at 28°C, and then the bacterial cells were separated by centrifugation and resuspended to an OD_600_ of 0.75 in infiltration buffer (10 mmol/L MES, 10 mmol/L MgCl_2_, 150 μmol/L acetosyringone). Then the *A. tumefaciens* strains carrying the GFP constructs or empty GFP vector (as control) were transformed into tobacco leaves (*N. benthamiana*) together with an ER-localization marker ER-mCherry [23]. After injection for 3 days, the fluorescence signals were observed and photographed under confocal laser scanning microscope.

### Transient overexpression of CitFNSII-1 and CitFNSII-2 in citrus fruit

The transient overexpression assay was conducted following a previous method [40] with several adjustments. To be specific, the CDSs of *CitFNSIIs* were respectively inserted into the pBI121 vector. The CitFNSII-1-pBI121 and CitFNSII-2-pBI121 recombinants as well as the empty vector (as control) were respectively transferred into *A. tumefaciens* strain EHA105. The transformants carrying the recombinant vectors and the empty vector were primarily grown in LB Broth at 28°C, then were centrifugated and resuspended according to the description in subcellular localization. The suspensions containing the target genes and the control were separately injected into the peels of mature ‘Ponkan’ fruit on opposite sides as described in MeSA treatment. After infiltration, the fruits were stored in the dark for 24 h and then under a 16 h/8 h (light/dark) photoperiod for 3 days until sampling. PMFs content and transcription levels of *CitFNSII-1* and *CitFNSII-2* were measured as described above. Primers used in the construction of *CitFNSII-1*-pBI121 and *CitFNSII-2*-pBI121 and for CDS region RT–PCR are listed in Table S1 (see online supplementary material).

### TRV-mediated VIGS in citrus

The TRV-mediated VIGS in citrus was conducted consulting the previous methods [54], with slight modification. A 279-bp length sequence, highly conservative in both *CitFNSII-1* and *CitFNSII-2* (99% similarity in two genes)*,* was amplified and inserted into the TRV2 vector, to get *CitFNSII*-TRV2 constructs. The TRV1 and the *CitFNSII*-TRV2 recombinants were separately converted into *A. tumefaciens* strain EHA105. The *Agrobacterium* cells carrying TRV1 and *CitFNSII*-TRV2 constructs were grown in liquid LB medium to an OD_600_ = 0.8, then followed by centrifugation and resuspension in the infiltration buffer containing 10 mmol/L MES, 10 mmol/L MgCl_2_, and 200 μmol/L acetosyringone. The whole germinated seedlings of ‘Ponkan’ were infiltrated by a mixture of resuspended bacteria carrying TRV1 and *CitFNSII*-TRV2 constructs in 1:1 ratio through the vacuum method, and the seedlings infiltrated by a mixture of resuspended bacteria carrying TRV1 and the empty TRV2 vector were used as control. The seedlings then were cultivated in the dark for 3 days, and turned to light growth (16 h/8 h light/dark) until they took root before they were moved to an artificial climate chamber for 1 month. The PMF contents and transcript levels of *CitFNSII-1* and *CitFNSII-2* in the aerial parts were measured as mentioned above. The measurement of PMF contents and gene expression in each VIGS-positive plant and each control plant were performed for three technical replicates. Primers used in the construction of CitFNSIIs-TRV2 and for RT–PCR are presented in Table S1 (see online supplementary material).

### Statistics analysis

All experiments were repeated for at least three replicates and the data were presented as mean values and standard errors (SE). Origin 2021 was used to construct waterfall figures; Graphpad prism version 8 was used to plot other figures; ChemBioDraw Ultra version 20.0 was used to generate chemical structural formula. Data analysis was conducted using one-way ANOVA followed Duncan’s multiple range test and Student’s *t*-test. SPSS version 26 (Statistical Product and Service Solutions, Chicago, IL, USA) was used for significance analysis with a confidence level of 95.0% (^*^P < 0.05), 99.0% (^**^P < 0.01), 99.9% (^***^P < 0.001) or 99.99% (^****^P < 0.0001).

## Acknowledgments

This research was supported by the China Postdoctoral Science Foundation (2021 M700124 and 2021 M692845), the National Natural Science Foundation of China (32072132), and the Fundamental Research Funds for the Central Universities (K20220104).

## Author contributions

C.S., C.Zhao and J.Z. designed the project. J.Z. performed the experiments with assistance from C.Zhao, X.L., Z.L., Q.G., and C.Zhou. J.Z. and C.Zhao analysed the data and drafted the manuscript. Y.L. conducted the molecular docking analysis. Y.W. helped with the LC–MS analysis. L.L. and D.W. provided plant materials for this study. C.S. and J.C. improved the draft. All authors approved this article.

## Data availability

The raw data of transcriptome are available at GenBank. The accession numbers are PRJNA923786 and PRJNA924350. Other data presented in the research are all comprised in this article.

## Conflict of interest statement

All authors declare that they have no conflict of interest.

## Supplementary data

Supplementary data is available at *Horticulture Research* online.

## Supplementary Material

Web_Material_uhad113

## References

[1] Wang SC , Yang C, Tu H et al. Characterization and metabolic diversity of flavonoids in citrus species. Sci Rep. 2017;7:10549. 28874745 10.1038/s41598-017-10970-2 PMC5585201

[2] Sun ZG , Li ZN, Zhang JM et al. Recent developments of flavonoids with various activities. Curr Top Med Chem. 2022;22:305–29. 35040404 10.2174/1568026622666220117111858

[3] Abad-Garcia B , Garmon-Lobato S, Belen Sanchez-Ilarduya M et al. Polyphenolic contents in citrus fruit juices: authenticity assessment. Eur Food Res Technol. 2014;238:803–18.

[4] Berim A , Gang DR. Methoxylated flavones: occurrence, importance, biosynthesis. Phytochem Rev. 2016;15:363–90.

[5] Liu XJ , Zhao CN, Gong Q et al. Characterization of a caffeoyl-CoA O-methyltransferase-like enzyme involved in biosynthesis of polymethoxylated flavones in *Citrus reticulata*. J Exp Bot. 2020;71:3066–79. 32182355 10.1093/jxb/eraa083 PMC7475179

[6] Peng ZX , Zhang HP, Li WY et al. Comparative profiling and natural variation of polymethoxylated flavones in various citrus germplasms. Food Chem. 2021;354:129499. 33752115 10.1016/j.foodchem.2021.129499

[7] Wang Y , Qian J, Cao JP et al. Antioxidant capacity, anticancer ability and flavonoids composition of 35 citrus (*Citrus reticulata* Blanco) varieties. Molecules. 2017;22:20. 10.3390/molecules22071114 PMC6152254 28678176

[8] Wang Y , Liu XJ, Chen JB et al. Citrus flavonoids and their antioxidant evaluation. Crit Rev Food Sci Nutr. 2022;62:3833–54. 33435726 10.1080/10408398.2020.1870035

[9] Martens S , Mithofer A. Flavones and flavone synthases. Phytochemistry. 2005;66:2399–2407. 16137727 10.1016/j.phytochem.2005.07.013

[10] Wei S , Xiang Y, Zhang Y et al. The unexpected flavone synthase-like activity of polyphenol oxidase in tomato. Food Chem. 2022;377:131958. 34990951 10.1016/j.foodchem.2021.131958

[11] Martens S , Forkmann G, Matern U et al. Cloning of parsley flavone synthase I. Phytochemistry. 2001;58:43–6. 11524111 10.1016/s0031-9422(01)00191-1

[12] Falcone Ferreyra ML , Emiliani J, Jose Rodriguez E et al. The identification of maize and Arabidopsis type I FLAVONE SYNTHASEs links flavones with hormones and biotic interactions. Plant Physiol. 2015;169:1090–107. 26269546 10.1104/pp.15.00515 PMC4587447

[13] Lee YJ , Kim JH, Kim BG et al. Characterization of flavone synthase I from rice. BMB Rep. 2008;41:68–71. 18304453 10.5483/bmbrep.2008.41.1.068

[14] Li H , Li D, Yang Z et al. Flavones produced by mulberry flavone synthase type I constitute a defense line against the ultraviolet-B stress. Plants-Basel. 2020;9:215. 32045991 10.3390/plants9020215 PMC7076714

[15] Wang HJ , Liu SH, Wang TL et al. The moss flavone synthase I positively regulates the tolerance of plants to drought stress and UV-B radiation. Plant Sci. 2020;298:110591. 32771149 10.1016/j.plantsci.2020.110591

[16] Ayabe S-i , Akashi T. Cytochrome P450s in flavonoid metabolism. Phytochem Rev. 2006;5:271–82.

[17] Lam PY , Zhu FY, Chan WL et al. Cytochrome P450 93G1 is a flavone synthase II that channels flavanones to the biosynthesis of Tricin O-linked conjugates in rice. Plant Physiol. 2014;165:1315–27. 24843076 10.1104/pp.114.239723 PMC4081339

[18] Fliegmann J , Furtwangler K, Malterer G et al. Flavone synthase II (CYP93B16) from soybean (*Glycine max* L.). Phytochemistry. 2010;71:508–14. 20132953 10.1016/j.phytochem.2010.01.007

[19] Zhang J , Subramanian S, Zhang Y et al. Flavone synthases from *Medicago truncatula* are flavanone-2-hydroxylases and are important for nodulation. Plant Physiol. 2007;144:741–51. 17434990 10.1104/pp.106.095018 PMC1914186

[20] Du YG , Chu H, Wang MF et al. Identification of flavone phytoalexins and a pathogen-inducible flavone synthase II gene (SbFNSII) in sorghum. J Exp Bot. 2010;61:983–94. 20007684 10.1093/jxb/erp364 PMC2826645

[21] Righini S , Rodriguez EJ, Berosich C et al. Apigenin produced by maize flavone synthase I and II protects plants against UV-B-induced damage. Plant Cell Environ. 2019;42:495–508. 30160312 10.1111/pce.13428

[22] Akashi T , Aoki T, Ayabe SI. Identification of a cytochrome P450 cDNA encoding (2S)-favanone 2-hydroxylase of licorice (*Glycyrrhiza echinata* L.; Fabaceae) which represents licodione synthase and favone synthase II. FEBS Lett. 1998;431:287–90. 9708921 10.1016/s0014-5793(98)00781-9

[23] Fujino N , Tenma N, Waki T et al. Physical interactions among flavonoid enzymes in snapdragon and torenia reveal the diversity in the flavonoid metabolon organization of different plant species. Plant J. 2018;94:372–92. 29421843 10.1111/tpj.13864

[24] Zhao Q , Zhang Y, Wang G et al. A specialized flavone biosynthetic pathway has evolved in the medicinal plant, *Scutellaria baicalensis*. Sci Adv. 2016;2:15. 10.1126/sciadv.1501780 PMC4846459 27152350

[25] Akashi T , Fukuchi-Mizutani M, Aoki T et al. Molecular cloning and biochemical characterization of a novel cytochrome P450, flavone synthase II, that catalyzes direct conversion of flavanones to flavones. Plant Cell Physiol. 1999;40:1182–6. 10635120 10.1093/oxfordjournals.pcp.a029505

[26] Deguchi A , Ohno S, Hosokawa M et al. Endogenous post-transcriptional gene silencing of flavone synthase resulting in high accumulation of anthocyanins in black dahlia cultivars. Planta. 2013;237:1325–35. 23389674 10.1007/s00425-013-1848-6

[27] Ueyama Y , Suzuki K, Fukuchi-Mizutani M et al. Molecular and biochemical characterization of torenia flavonoid 3'-hydroxylase and flavone synthase II and modification of flower color by modulating the expression of these genes. Plant Sci. 2002;163:253–63.

[28] Zhou LJ , Geng ZQ, Wang YX et al. A novel transcription factor CmMYB012 inhibits flavone and anthocyanin biosynthesis in response to high temperatures in chrysanthemum. Horticulture Research. 2021;8:248. 34848687 10.1038/s41438-021-00675-z PMC8633327

[29] Premathilake AT , Ni JB, Shen JQ et al. Transcriptome analysis provides new insights into the transcriptional regulation of methyl jasmonate-induced flavonoid biosynthesis in pear calli. BMC Plant Biol. 2020;20:388. 32842960 10.1186/s12870-020-02606-x PMC7446162

[30] Sun H , Cui HT, Zhang JJ et al. Gibberellins inhibit flavonoid biosynthesis and promote nitrogen metabolism in *Medicago truncatula*. Int J Mol Sci. 2021;22:9291. 10.3390/ijms22179291 PMC8431309 34502200

[31] Fariborz H , Asghar R, Guillen F et al. Blood oranges maintain bioactive compounds and nutritional quality by postharvest treatments with gamma-aminobutyric acid, methyl jasmonate or methyl salicylate during cold storage. Food Chem. 2020;306:125634. 31614291 10.1016/j.foodchem.2019.125634

[32] Ortuno A , Diaz L, Alvarez N et al. Comparative study of flavonoid and scoparone accumulation in different citrus species and their susceptibility to *Penicillium digitatum*. Food Chem. 2011;125:232–9.

[33] Almada-Ruiz E , Martinez-Tellez MA, Hernandez-Alamos MM et al. Fungicidal potential of methoxylated flavones from citrus for *in vitro* control of *Colletotrichum gloeosporioides*, causal agent of anthracnose disease in tropical fruits. Pest Manag Sci. 2003;59:1245–9. 14620052 10.1002/ps.747

[34] Silva CCD , Shimo HM, de Felicio R et al. Structure-function relationship of a citrus salicylate methylesterase and role of salicylic acid in citrus canker resistance. Sci Rep. 2019;9:3901. 30846791 10.1038/s41598-019-40552-3 PMC6405950

[35] Deng B , Wang WJ, Ruan CQ et al. Involvement of CsWRKY70 in salicylic acid-induced citrus fruit resistance against *Penicillium digitatum*. Horticulture Research. 2020;7:157. 33082964 10.1038/s41438-020-00377-y PMC7527965

[36] Yamamoto R , Ma G, Zhang LC et al. Effects of salicylic acid and methyl Jasmonate treatments on flavonoid and carotenoid accumulation in the juice sacs of Satsuma mandarin in vitro. Appl Sci (Basel). 2020;10:13.

[37] Li X , Zhang L-P, Zhang L et al. Methyl salicylate enhances flavonoid biosynthesis in tea leaves by stimulating the phenylpropanoid pathway. Molecules. 2019;24:362. 30669582 10.3390/molecules24020362 PMC6359712

[38] Tian SL , Yang YY, Wu T et al. Functional characterization of a flavone synthase that participates in a kumquat flavone Metabolon. Front Plant Sci. 2022;13:13. 10.3389/fpls.2022.826780 PMC8924551 35310637

[39] Liu X , Gong Q, Zhao C et al. Genome-wide analysis of cytochrome P450 genes in *Citrus clementina* and characterization of a CYP gene encoding flavonoid 3′-hydroxylase. Horticulture Research. 2023;10:uhac283. 36818367 10.1093/hr/uhac283 PMC9930397

[40] Zhao CN , Liu XJ, Gong Q et al. Three AP2/ERF family members modulate flavonoid synthesis by regulating type IV chalcone isomerase in citrus. Plant Biotechnol J. 2021;19:671–88. 33089636 10.1111/pbi.13494 PMC8051604

[41] Wu J , Wang XC, Liu Y et al. Flavone synthases from *Lonicera japonica* and *L. macranthoides* reveal differential flavone accumulation. Sci Rep. 2016;6:19245. 26754912 10.1038/srep19245 PMC4709722

[42] Brito A , Ramirez JE, Areche C et al. HPLC-UV-MS profiles of phenolic compounds and antioxidant activity of fruits from three citrus species consumed in northern Chile. Molecules. 2014;19:17400–21. 25356563 10.3390/molecules191117400 PMC6271594

[43] Fayek NM , Farag MA, Abdel Monem AR et al. Comparative metabolite profiling of four citrus Peel cultivars via ultra-performance liquid chromatography coupled with Quadrupole-time-of-flight-mass spectrometry and multivariate data analyses. J Chromatogr Sci. 2019;57:349–60. 30796772 10.1093/chromsci/bmz006

[44] Ledesma-Escobar CA , Priego-Capote F, Olvera VJR et al. Targeted analysis of the concentration changes of phenolic compounds in Persian lime (*Citrus latifolia*) during fruit growth. J Agric Food Chem. 2018;66:1813–20. 29400054 10.1021/acs.jafc.7b05535

[45] Seoka M , Ma G, Zhang LC et al. Expression and functional analysis of the nobiletin biosynthesis-related gene CitOMT in citrus fruit. Sci Rep. 2020;10:11. 32943728 10.1038/s41598-020-72277-z PMC7498457

[46] Ramabulana A-T , Steenkamp PA, Madala NE et al. Profiling of altered metabolomic states in *Bidens pilosa* leaves in response to treatment by methyl jasmonate and methyl salicylate. Plan Theory. 2020;9:1275. 10.3390/plants9101275 PMC7601133 32992670

[47] Gondor OK , Janda T, Soos V et al. Salicylic acid induction of flavonoid biosynthesis pathways in wheat varies by treatment. Front Plant Sci. 2016;7:12. 27733857 10.3389/fpls.2016.01447 PMC5039175

[48] Kiefer E , Heller W, Ernst D. A simple and efficient protocol for isolation of functional RNA from plant tissues rich in secondary metabolites. Plant Mol Biol Report. 2000;18:33–9.

[49] Pillitteri LJ , Lovatt CJ, Walling LL. Isolation and characterization of LEAFY and APETALA1 homologues from *Citrus sinensis* L. Osbeck ‘Washington. J Am Soc Hortic Sci. 2004;129:846–56.

[50] Kumar S , Stecher G, Li M et al. MEGA X: molecular evolutionary genetics analysis across computing platforms. Mol Biol Evol. 2018;35:1547–9. 29722887 10.1093/molbev/msy096 PMC5967553

[51] Liu Y , Wang R, Ren C et al. Two myricetin-derived flavonols from *Morella rubra* leaves as potent α-glucosidase inhibitors and structure-activity relationship study by computational chemistry. Oxidative Med Cell Longev. 2022;2022:9012943. 10.1155/2022/9012943 PMC9042601 35498126

[52] Trott O , Olson AJ. AutoDock Vina: improving the speed and accuracy of docking with a new scoring function, efficient optimization, and multithreading. J Comput Chem. 2010;31:455–61. 19499576 10.1002/jcc.21334 PMC3041641

[53] Szymanski EP , Kerscher O. Budding yeast protein extraction and purification for the study of function, interactions, and post-translational modifications. JoVE. 2013;e50921. 24300101 10.3791/50921 PMC3968972

[54] Wang FS , Wang M, Liu XN et al. Identification of putative genes involved in limonoids biosynthesis in citrus by comparative transcriptomic analysis. Front Plant Sci. 2017;8:12. 28553308 10.3389/fpls.2017.00782 PMC5427120

