# Supplementary material for: Functional characterization of two flavone synthase II members in citrus
Source: Hortic Res. 2023 May 31;10(7):uhad113. doi: 10.1093/hr/uhad113 (PMC10419818; doi:10.1093/hr/uhad113)
Supplement: Web_Material_uhad113 [file web_material_uhad113.zip › Supplemental data-R3-v3.docx]

| **Gene** | **Forward primer （5‘ to 3’）** | **Reverse primer （5‘ to 3’）** |
| --- | --- | --- |
| CitFNSII（for cloning） | ATGACACTTCAACCACTGA | GCTCCTAGTATCCTAAATTAG |
| CitFNSII-1（for cloning from NHL, BTC, YR） | ATGACACTTCAACCACTGA | GTTGCATGCCAACACTGTATG |
| CitFNSII-2（for cloning from NHL, BTC, YR） | ATGACACTTCAACCACTGA | TTCTAGGGTGAGTTAACCAC |
| CitFNSII-1-pYES NT/C | AAGGTACCCGGATCCATGACACTTCAACCACTGAT | CCCTCTAGACTCGAGATTTAGGATACTAGGAGCACAAC |
| CitFNSII-2-pYES NT/C | GACGATAAGGTACCCGGATCCATGACACTTCAACCACTGATTTTTTAT | GAAGGGCCCTCTAGACTCGAGATTTACGATACTAGGAGCACAACGTG |
| CitFNSII-1-pBI121 | GGACTCTAGAGGATCCATGACACTTCAACCACTGATTTTT | GACCACCCGGGGATCCATTTAGGATACTAGGAGCACAACGT |
| CitFNSII-2-pBI121 | GGACTCTAGAGGATCCATGACACTTCAACCACTGATTT | GACCACCCGGGGATCCATTTACGATACTAGGAGCACAACGT |
| CitFNSIIs-TRV2 | GCCTCCATGGGGATCCATGACACTTCAACCACTGA | ATGCCCGGGCCTCGAGAAAAGTAACATCATTTGTTTTGAGA |
| CitFNSII-1-qPCR | TGTACCAGTTGCACGTTGTG | CACGCTGCATGGTAGGTTGA |
| CitFNSII-2-qPCR | CGATTGCTGCCATTTGGAACT | AGCTGCTAGAGTTGTCGGC |
| CitFNSII-1-qPCR (for transient overexpression) | CACAGATACTTCATCCACGTCA | CAAGCCTGTTTCTTCCCACAAC |
| CitFNSII-2-qPCR (for transient overexpression) | CGATTGCTGCCATTTGGAACT | AGCTGCTAGAGTTGTCGGC |
| Citrus β-actin-qPCR | CATCCCTCAGCACCTTCC | CCAACCTTAGCACTTCTCC |

**Supplemental Table 1 Primers used in this study**

| **Short name** | **Organism** |  | **Genebank acession number** |
| --- | --- | --- | --- |
| OsFNSII | *Oryza sativa* | CYP93G1 | Q0JFI2.1 |
| SbFNSII | *Sorghum bicolor* | CYP93G3 | XP_002461286.1 |
| ZmFNSII | *Zea mays* | CYP93G7 | XP_008663013.1 |
| GeFNSII | *Glycyrrhiza echinata* | CYP93B1 | P93149.1 |
| MtFNSII-1 | *Medicago truncatula* | CYP90B10 | XP_003622125.1 |
| MtFNSII-2 | *Medicago truncatula* | CYP90B11 | XP_003622129.1 |
| GmFNSII | *Glycine max* | CYP93B16 | ACV65037.1 |
| ObFNSII | *Ocimum basilicum* | CYP93B23 | AGF30365.1 |
| SbFNSII-1 | *Scutellaria baicalensis* | CYP93B24 | AMW91728.1 |
| SbFNSII-2 | *Scutellaria baicalensis* | CYP93B25 | AMW91729.1 |
| LjFNSII-1.1 | *Lonicera japonica* |  | KU127576.1 |
| LjFNSII-2.1 | *Lonicera japonica* |  | KU127578.1 |
| LmFNSII-1.1 | *L. macranthoides* |  | KU127580.1 |
| FcFNSII-1 | *Fortunella crassifolia* |  | sjg260860.1 |
| FcFNSII-2 | *Fortunella crassifolia* |  | sjg260830.1 |

**Supplemental Table 2** **Information of FNSIIs used to construct the phylogenetic tree**


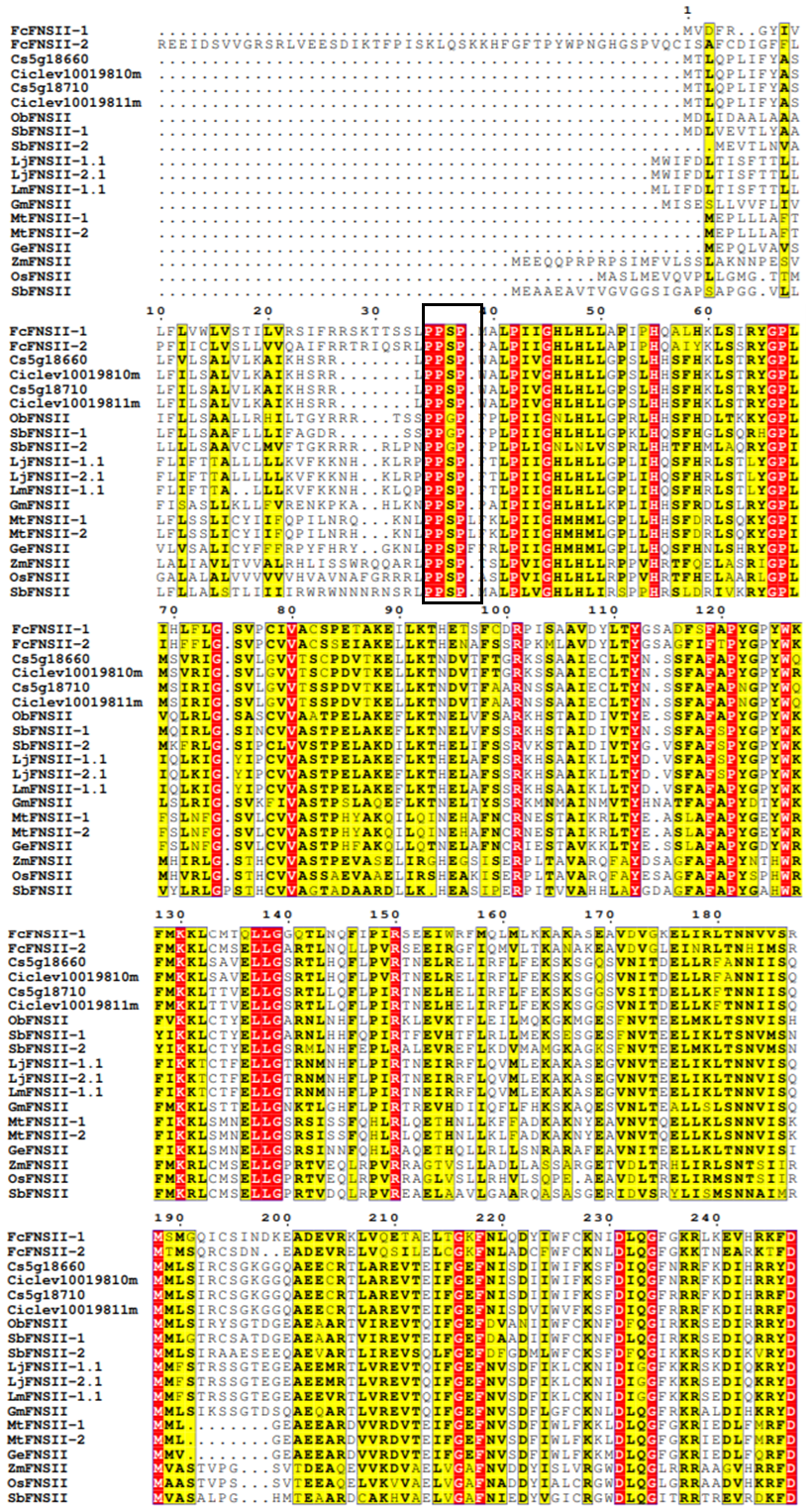


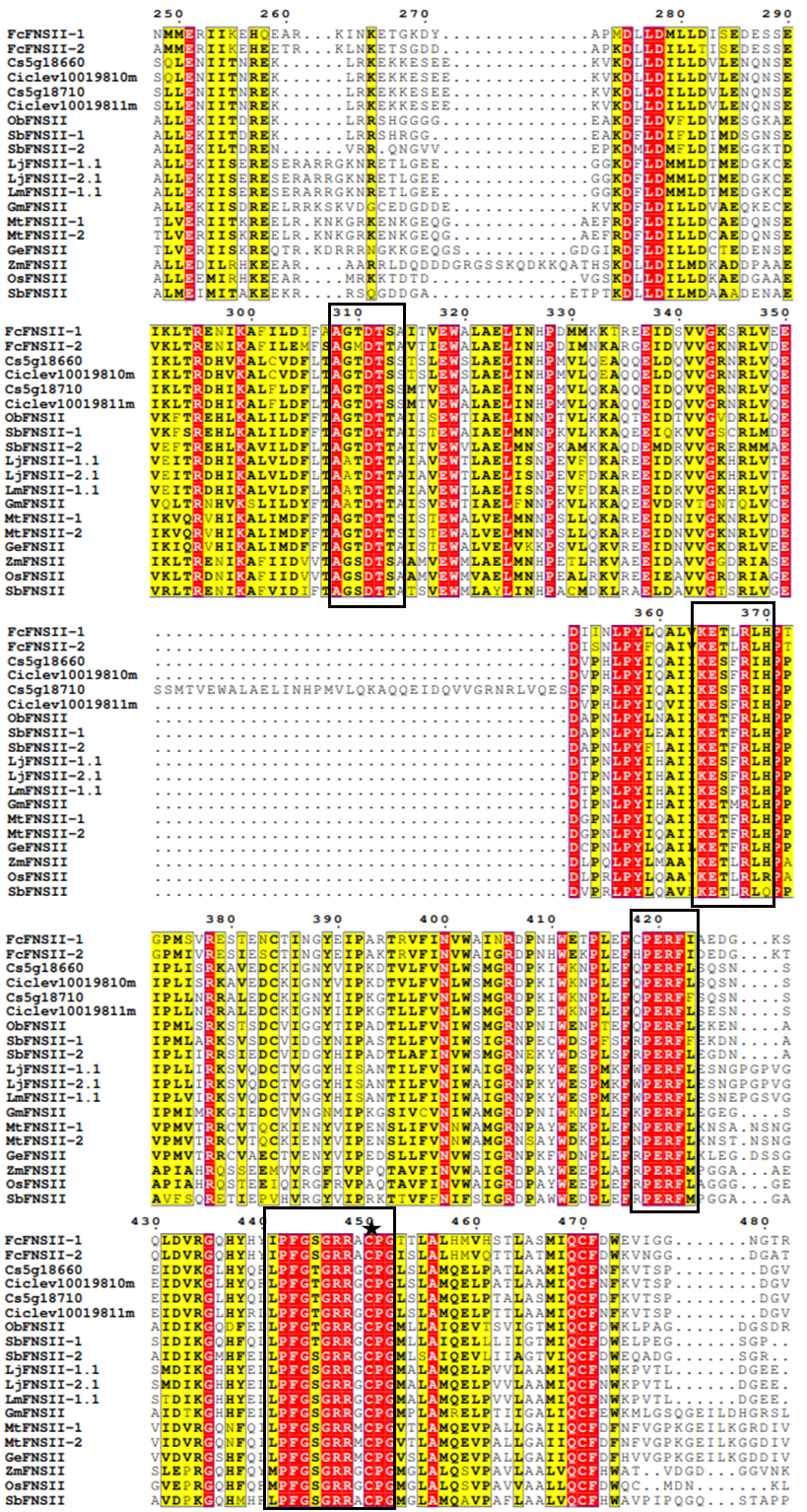


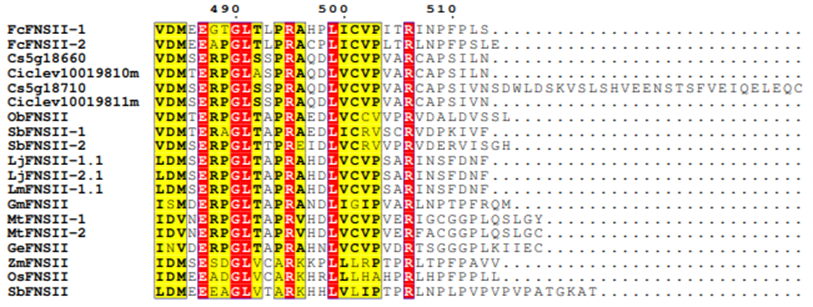


**Supplemental Figure 1** **The amino acid sequence alignment of CitFNSIIs with FNSIIs from other species**. OsFNSII (*Oryza sativa*), SbFNSII (*Sorghum bicolor*), ZmFNSII (*Zea mays*), GeFNSII (*Glycyrrhiza echinate*), MtFNSII-1 and MtFNSII-2 (*Medicago truncatula*), GmFNSII (*Glycine max*), SbFNSII-1 and SbFNSII-2 (*Scutellaria baicalensis*), LmFNSII-1.1 (*Lonicera macranthoides*), LjFNSII-1.1 and LjFNSII-2.1 (*Lonicera japonica*). The proline rich membrane hinge (RPPPSP), I-helix (AGTDTDS), E-R-R triade consisting of the K-helix consensus sequence (KESFR) and the consensus sequence (PERF), heme-binding domain (PFGTGRRGCPG) are boxed; these are conserved motifs among P450s. The cysteine in the heme-binding domain is marked by black stars and is conserved in all plant P450 sequences.


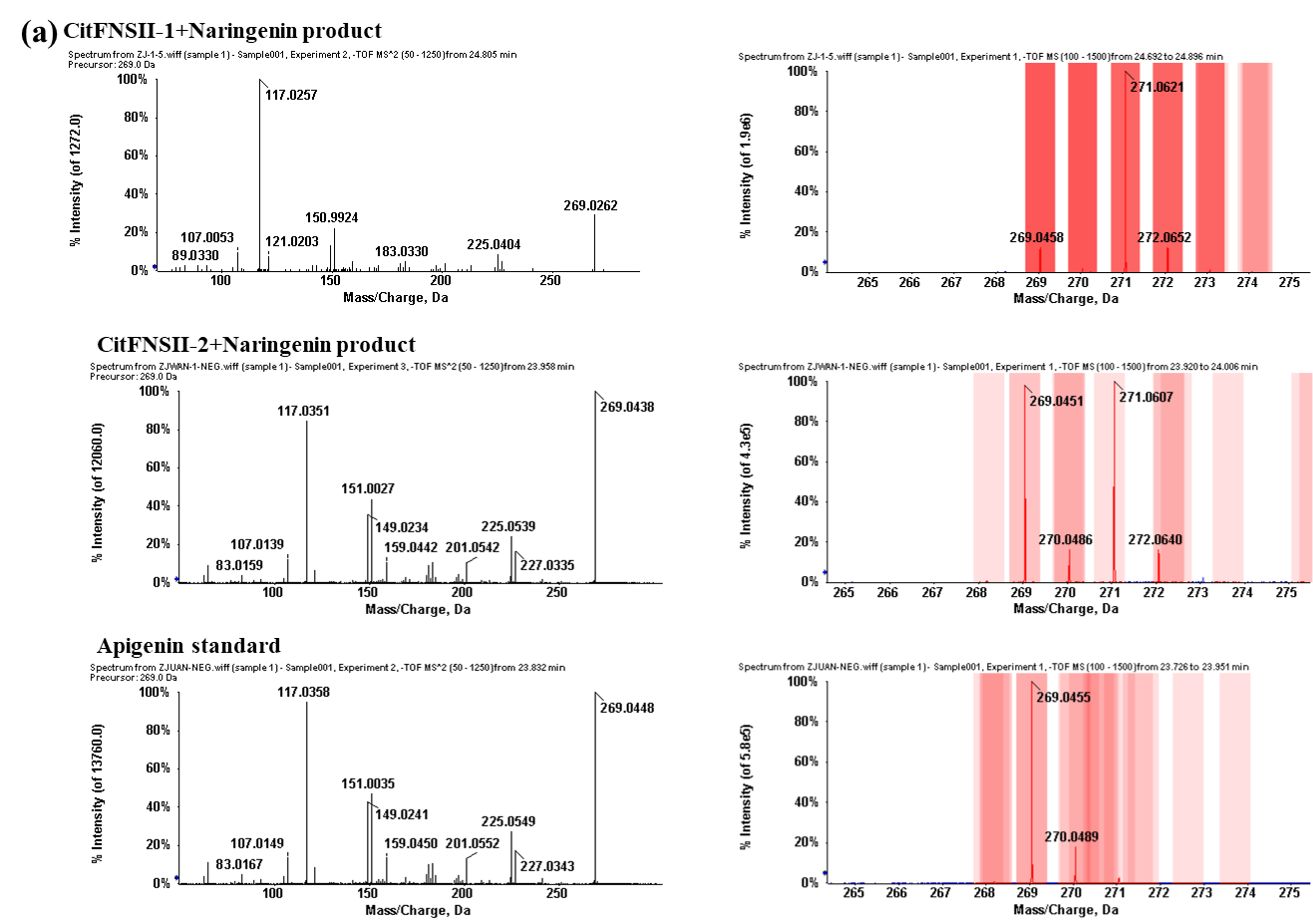


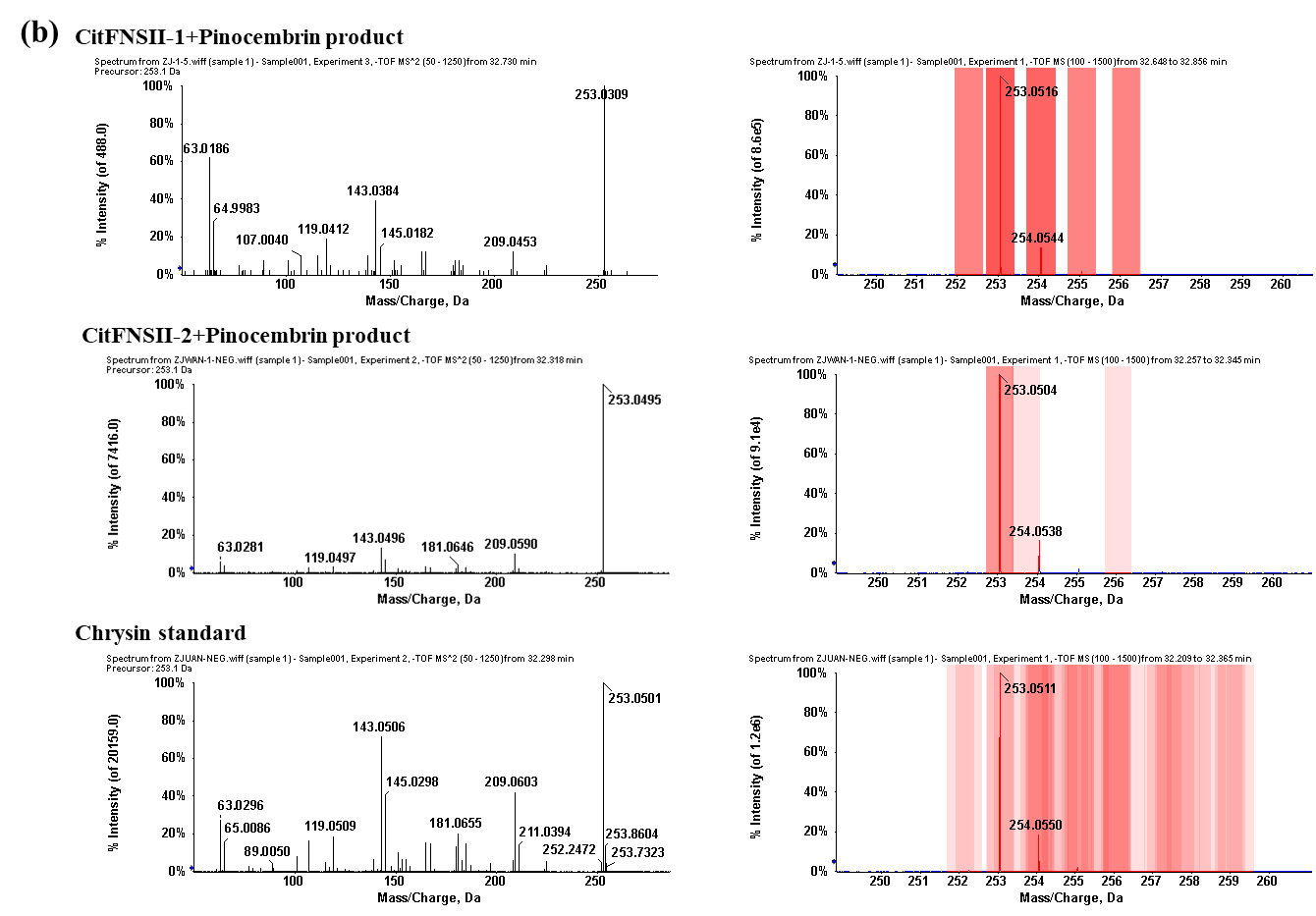


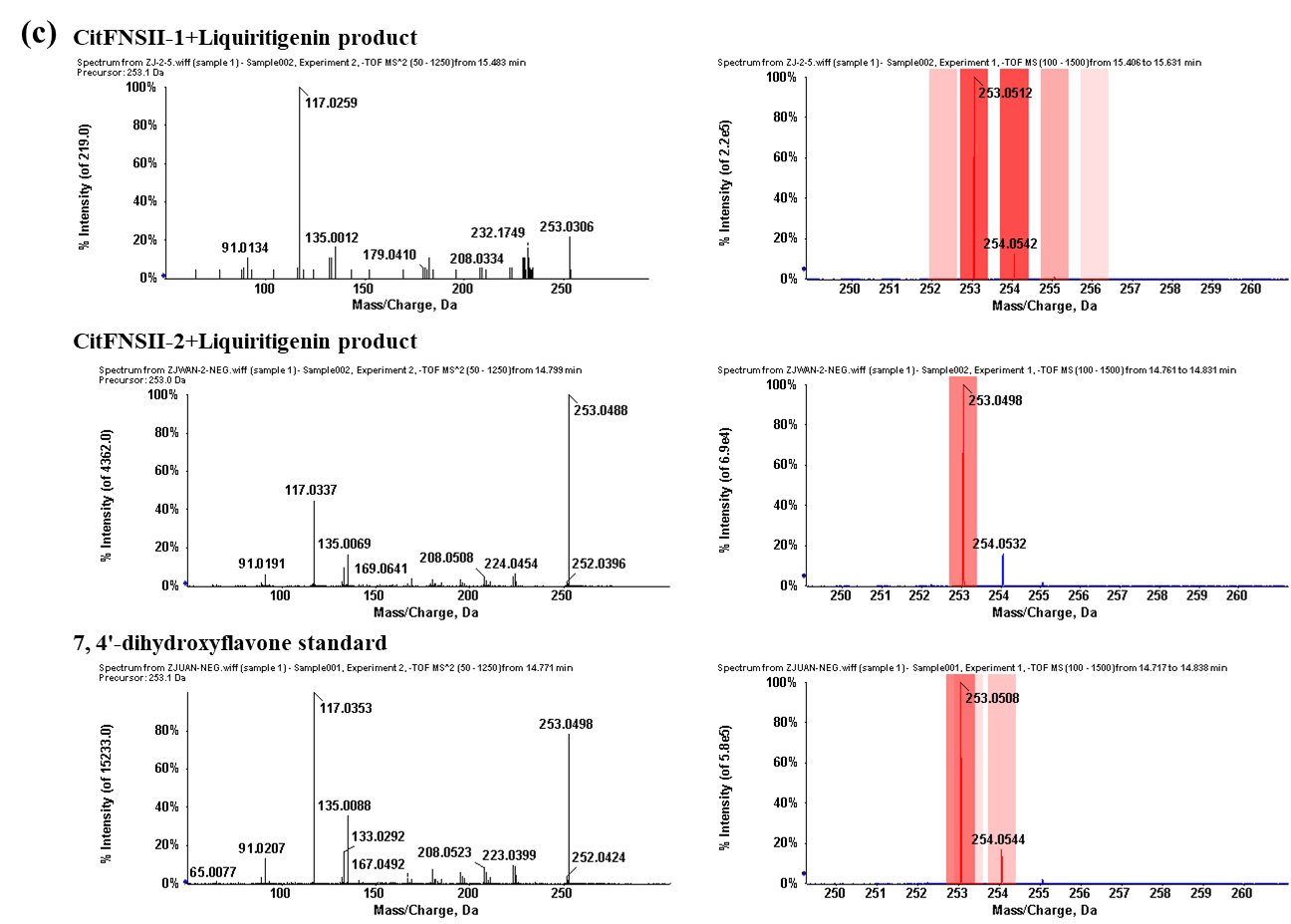


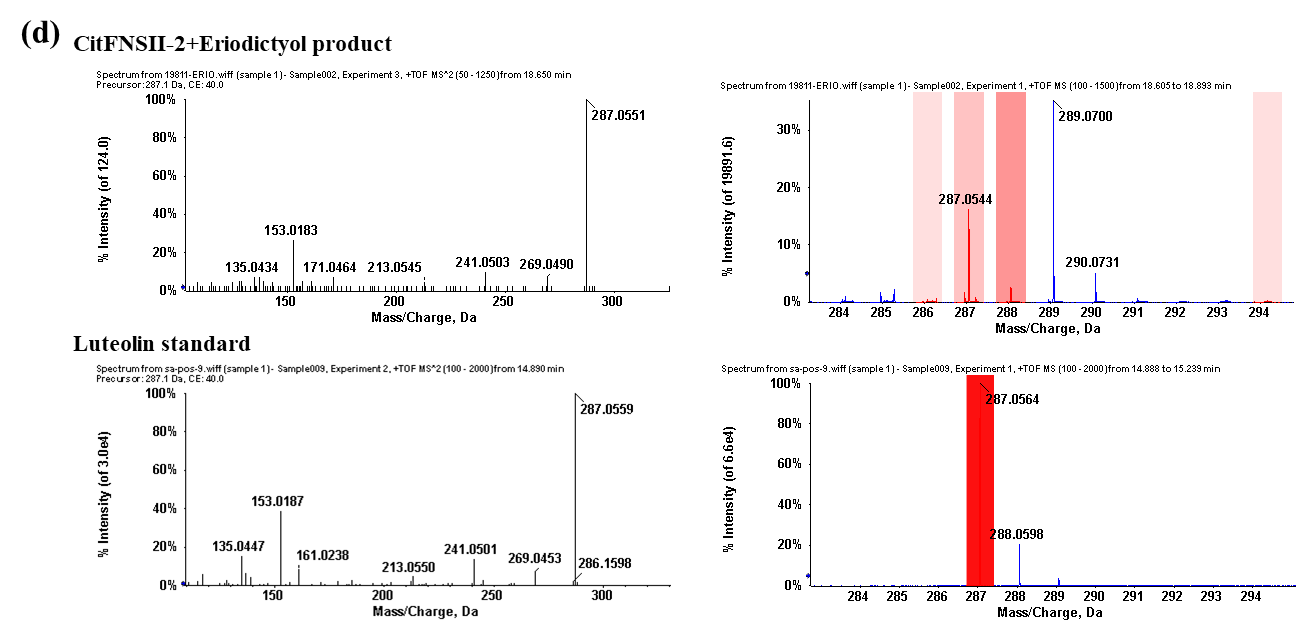


**Supplemental Figure 2** **MS/MS spectrometry of products generated by CitFNSIIs *in vivo* yeast** **and corresponding authentic standards.** (a) Naringenin incubated with recombinant CitFNSII-1 and CitFNSII-2 in yeast; (b) Pinocembrin incubated with recombinant CitFNSII-1 and CitFNSII-2 in yeast; (c) Liquiritigenin incubated with recombinant CitFNSII-1 and CitFNSII-2 in yeast. (d) Eriodictyol incubated with recombinant CitFNSII-2 in yeast. The MS/MS spectrums are listed at left side and the MS spectrums are listed at right side.

**(a)**


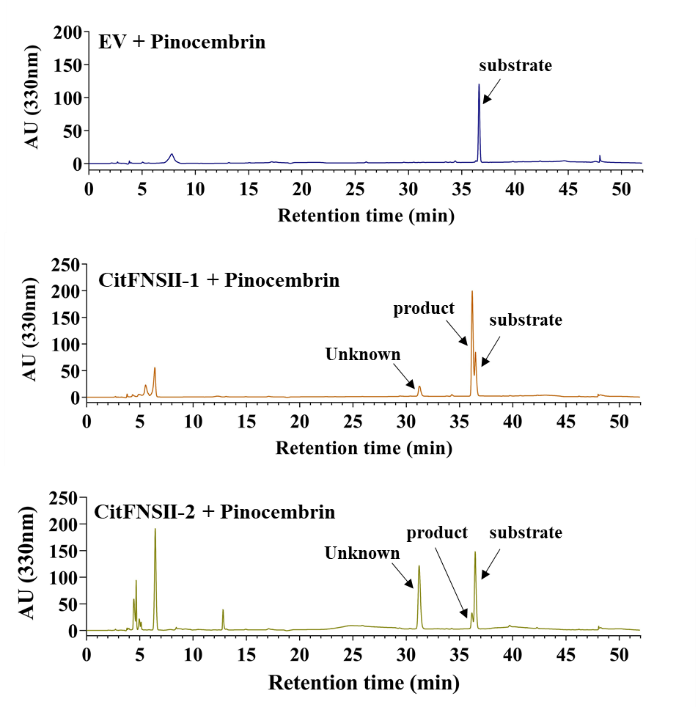


**(b)**


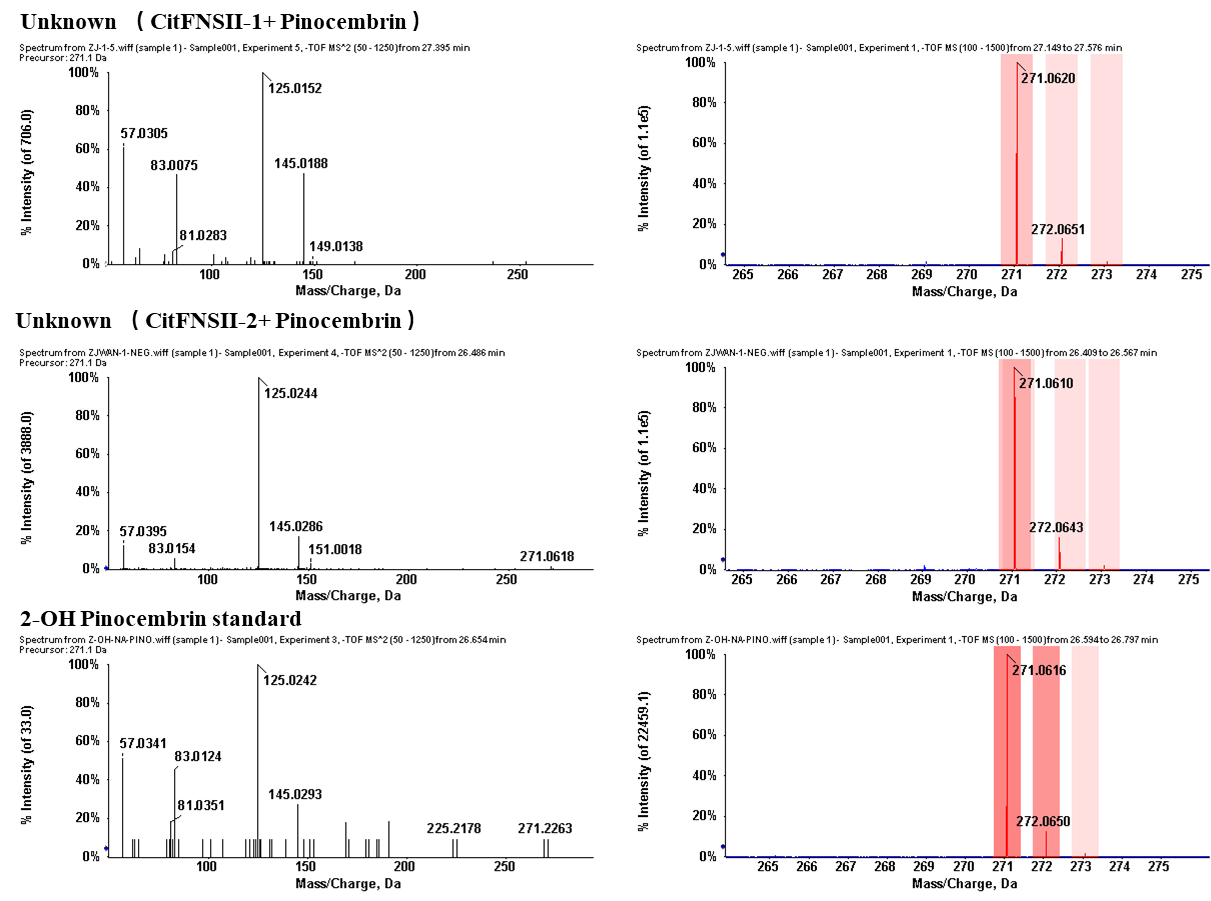


**Supplemental Figure 3** **Unknown product generated by the reaction of transformed yeast with pinocembrin.** (a) The HPLC chromatograms of extraction generated by the reaction of transformed yeast with pinocembrin. (b) MS/MS spectrometry of unknown products generated by CitFNSIIs *in vivo* yeast and corresponding authentic standard 2-OH pinocembrin. EV, Yeast transformed with empty pYES2 NT/C vector. The MS/MS spectrums are listed at left side and the MS spectrums are listed at right side.


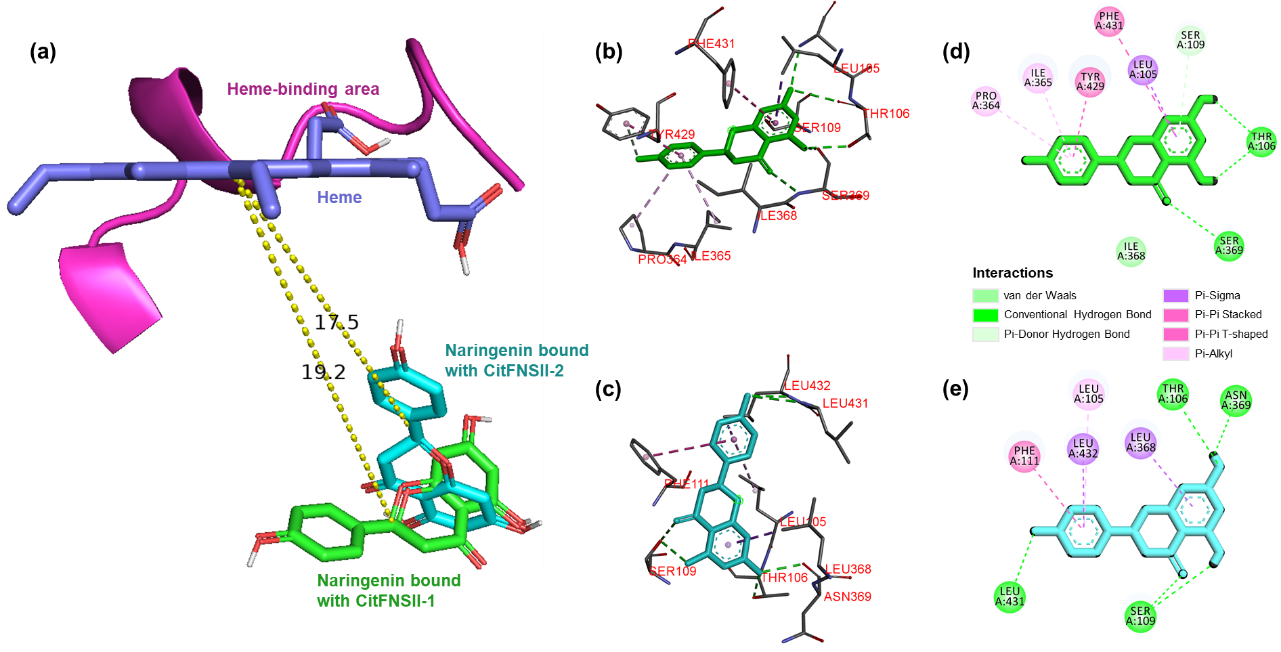


**Supplemental Figure 4 Molecular docking of naringenin binding with CitFNSII-1 and CitFNSII-2.** (a) Ligand modeling results indicate that naringenin binds in different orientations in CitFNSII-1 (green) and CitFNSII-2 (cyan). The 3D homology models of CitFNSII-1 and CitFNSII-2 were built based on the structures of 8E83_A (43.38% identity and 44.03% identity, respectively). The heme-binding area in CitFNSII-1 and CitFNSII-2 are coincident and marked in violet, the heme is marked in purple. (b-c) 3D model of detailed interactions of naringenin bound with key residues from CitFNSII-1 (b) and CitFNSII-2 (c). (d-e) 2D model of of naringenin bound with key residues from CitFNSII-1 (d) and CitFNSII-2 (e) and the interaction details.


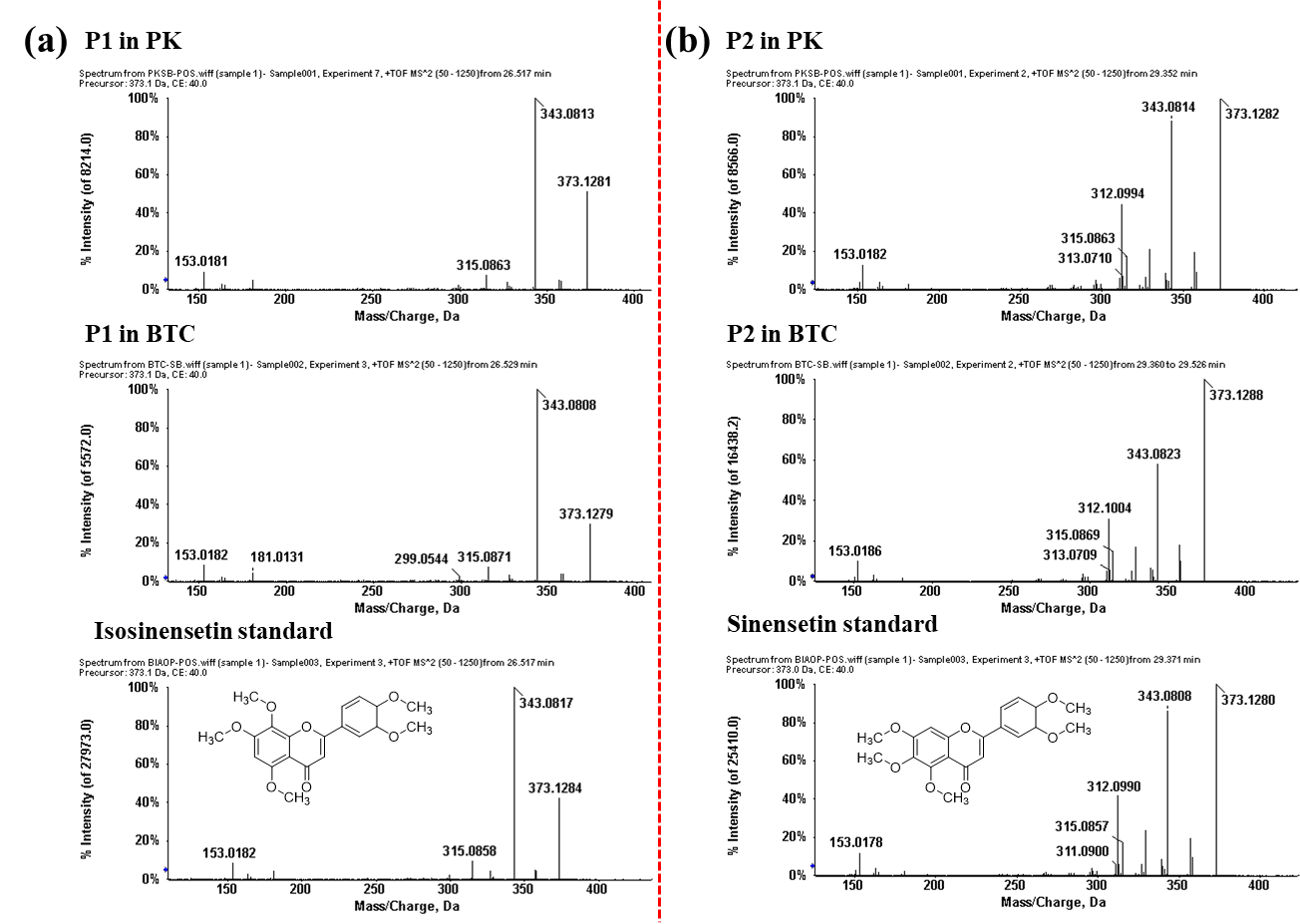

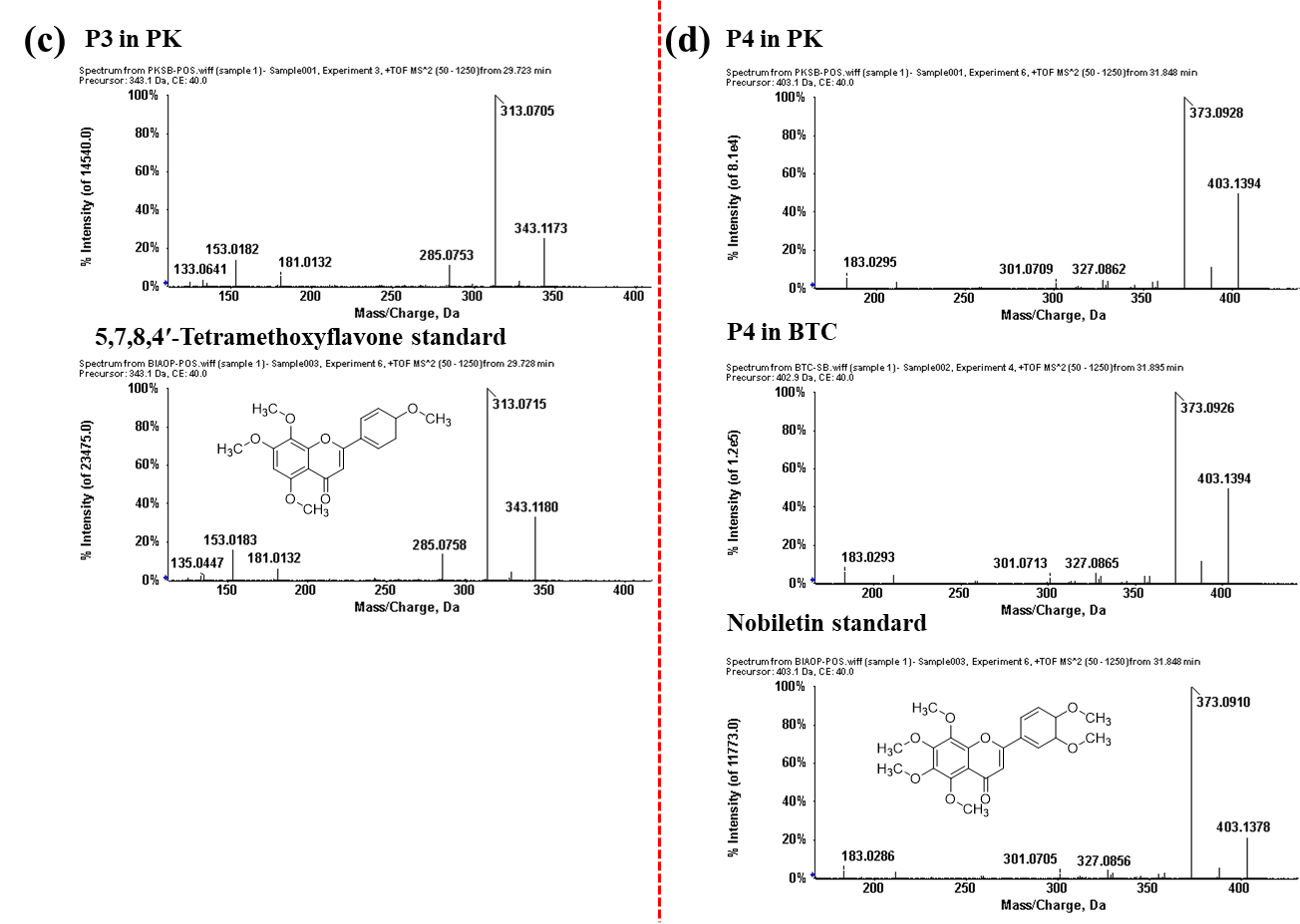


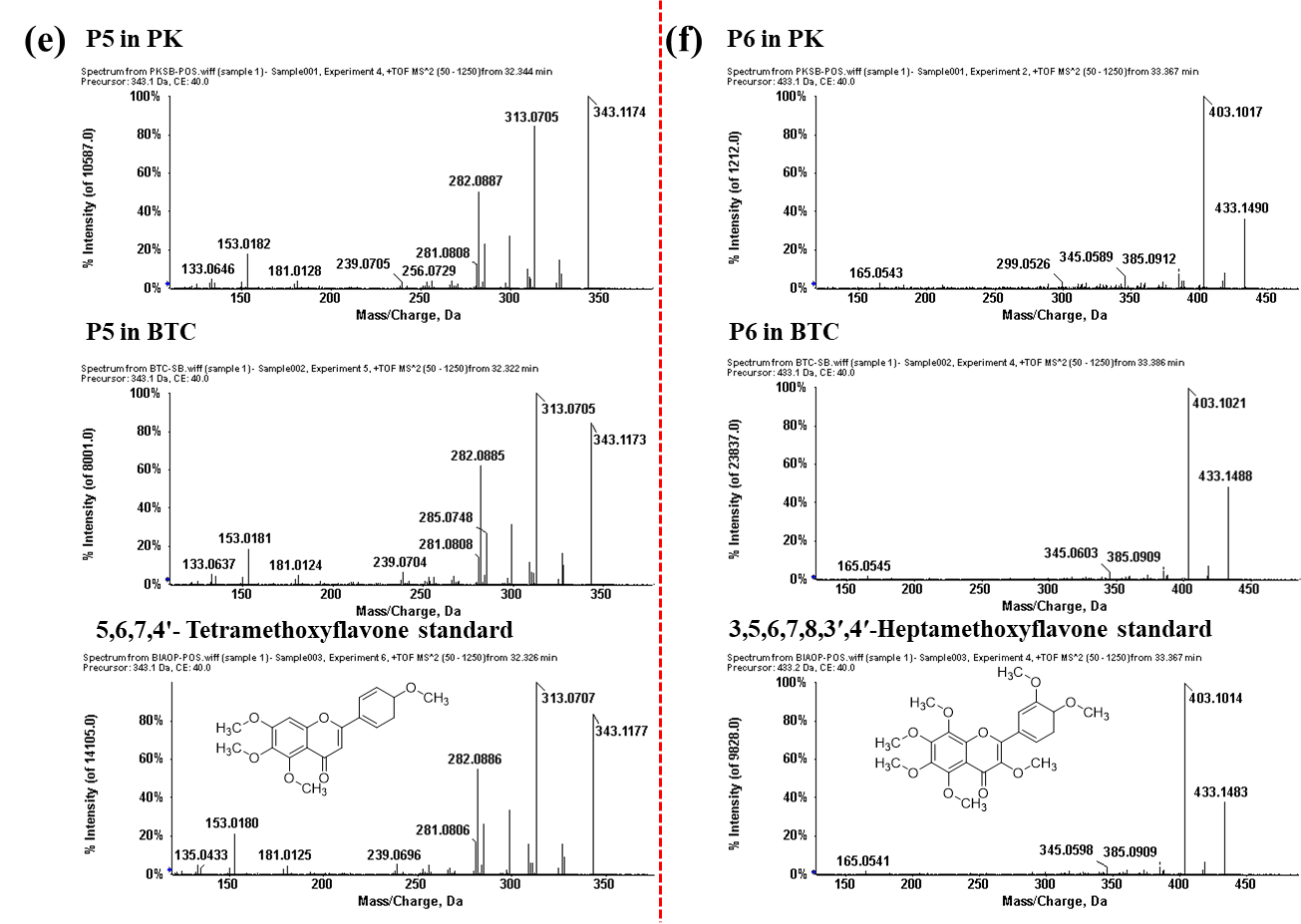


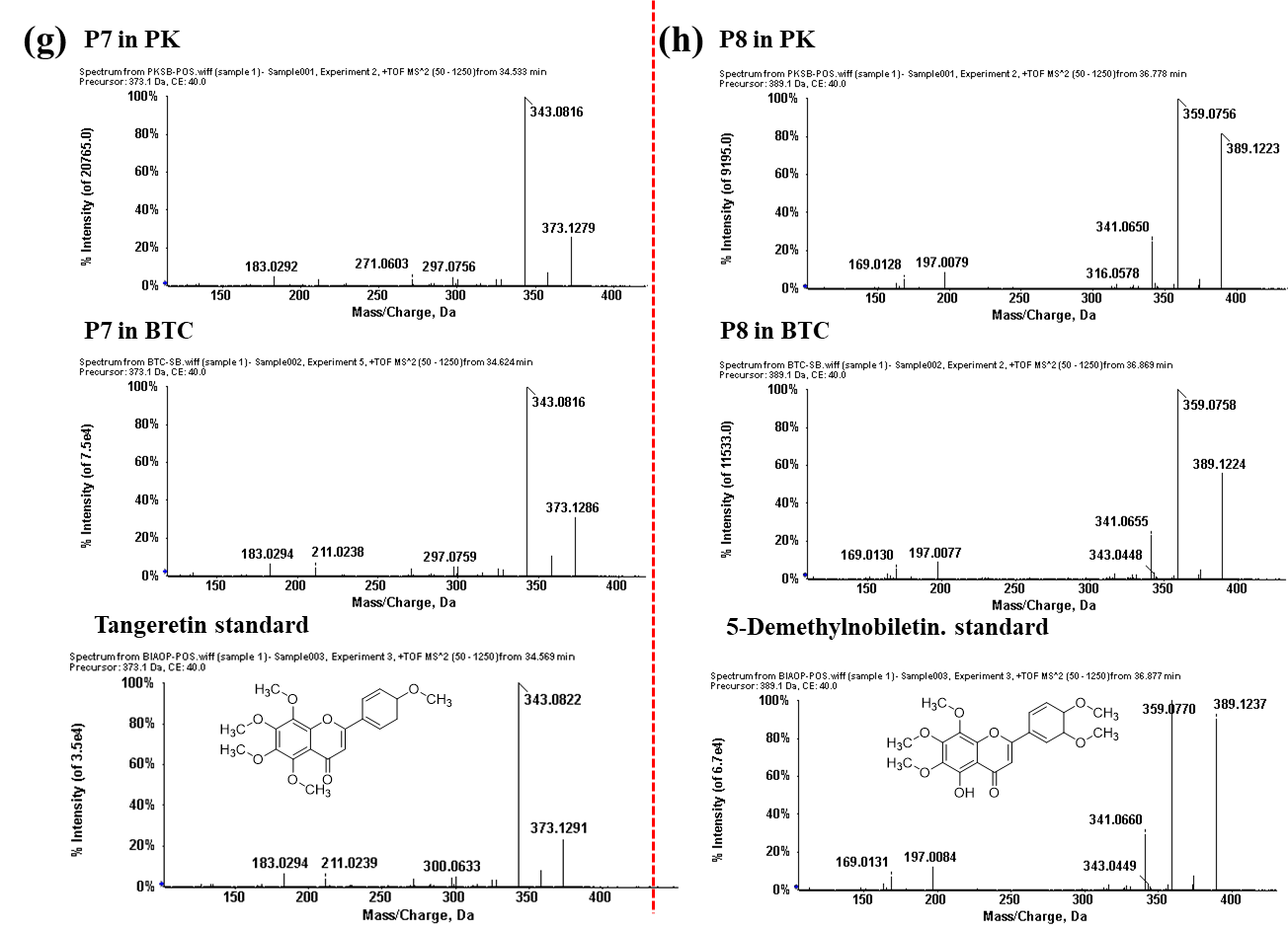


**Supplemental Figure 5 The LC-MS/MS of representative PMFs in flavedo of PK and BTC.** (a) P1, isosinensetin; (b) P2, sinensetin; (c) P3, 5,7,8,4′-tetramethoxyflavone; (d) P4, nobiletin; (e) P5, 5,6,7,4′-tetramethoxyflavone; (f) P6, 3,5,6,7,8,3′,4′-heptamethoxyflavone; (g) P7, tangeretin; (h) P8, 5-demethylnobiletin.


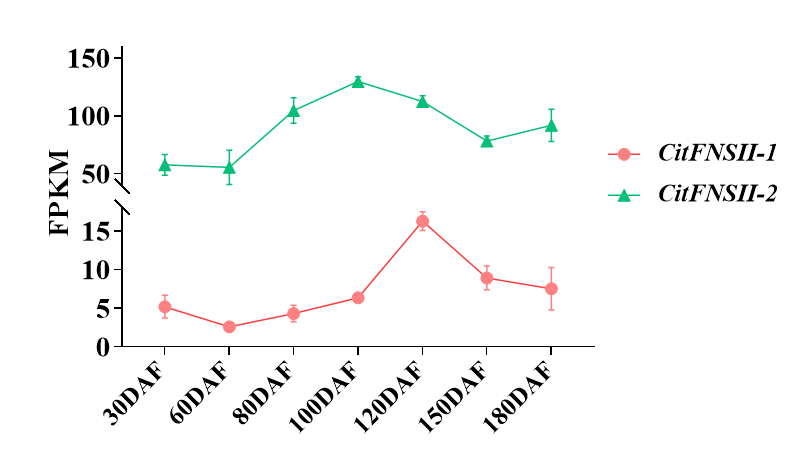


**Supplemental Figure** **6 Dynamics of *CitFNSII-1* and *CitFNSII-2* expression during fruit development of BTC.** DAF, days after flowering. These results are averages of FPKM values of three biological replicates.
